# Identification and verification of m7G-Related genes as biomarkers for prognosis of sarcoma

**DOI:** 10.3389/fgene.2023.1101683

**Published:** 2023-02-03

**Authors:** Haotian Qin, Weibei Sheng, Jian Weng, Guoqing Li, Yingqi Chen, Yuanchao Zhu, Qichang Wang, Yixiao Chen, Qi Yang, Fei Yu, Hui Zeng, Ao Xiong

**Affiliations:** ^1^ National & Local Joint Engineering Research Center of Orthopaedic Biomaterials, Peking University Shenzhen Hospital, Shenzhen, China; ^2^ Department of Bone & Joint Surgery, Peking University Shenzhen Hospital, Shenzhen, China; ^3^ Department of Medical Ultrasound, Peking University Shenzhen Hospital, Shenzhen, China

**Keywords:** m7G, sarcoma, prognostic model, tumor immune microenvironment, ceRNA regulatory network

## Abstract

**Background:** Increasing evidence indicates a crucial role for N7-methylguanosine (m7G) methylation modification in human disease development, particularly cancer, and aberrant m7G levels are closely associated with tumorigenesis and progression *via* regulation of the expression of multiple oncogenes and tumor suppressor genes. However, the role of m7G in sarcomas (SARC) has not been adequately evaluated.

**Materials and methods:** Transcriptome and clinical data were gathered from the TCGA database for this study. Normal and SARC groups were compared for the expression of m7G-related genes (m7GRGs). The expression of m7GRGs was verified using real-time quantitative PCR (RT-qPCR) in SARC cell lines. Then, differentially expressed genes (DEGs) were identified between high and low m7GRGs expression groups in SARC samples, and GO enrichment and KEGG pathways were evaluated. Next, prognostic values of m7GRGs were evaluated by Cox regression analysis. Subsequently, a prognostic model was constructed using m7GRGs with good prognostic values by Lasso regression analysis. Besides, the relationships between prognostic m7GRGs and immune infiltration, clinical features, cuproptosis-related genes, and antitumor drugs were investigated in patients with SARC. Finally, a ceRNA regulatory network based on m7GRGs was constructed.

**Results:** The expression of ten m7GRGs was higher in the SARC group than in the control group. DEGs across groups with high and low m7GRGs expression were enriched for adhesion sites and cGMP-PKG. Besides, we constructed a prognostic model that consists of EIF4A1, EIF4G3, NCBP1, and WDR4 m7GRGs for predicting the survival likelihood of sarcoma patients. And the elevated expression of these four prognostic m7GRGs was substantially associated with poor prognosis and elevated expression in SARC cell lines. Moreover, we discovered that these four m7GRGs expressions were negatively correlated with CD4^+^ T cell levels, dendritic cell level and tumor purity, and positively correlated with tumor mutational burden, microsatellite instability, drug sensitivity and cuproptosis-related genes in patients with sarcomas. Then, a triple regulatory network of mRNA, miRNA, and lncRNA was established.

**Conclusion:** The current study identified EIF4A1, EIF4G3, NCBP1, and WDR4 as prognostic genes for SARC that are associated with m7G.These findings extend our knowledge of m7G methylation in SARC and may guide the development of innovative treatment options.

## Introduction

Sarcoma (SARC) is a very heterogeneous malignant solid tumor with more than 100 subtypes (Fornaciari, 2018; Siegel et al., 2019). Sarcomas can occur at any age, but are more prevalent among teenagers and young adults (Reed et al., 2019). Standard therapies for sarcomas include surgery, radiation, and chemotherapy; nevertheless, these approaches had some limitations. Only a few kinds, such as osteosarcoma, Ewing’s sarcoma, and rhabdomyosarcoma were applicable to chemotherapy and targeted therapy (Harwood et al., 2015; Hiniker and Donaldson, 2015; Paprcka et al., 2022). The metastasis rate of sarcomas within 5 years is as high as 50%, and only 5% of patients with sarcoma metastases survived for 5 years (Brennan et al., 2014; Zhu et al., 2020), despite the fact that surgery and radiation can eliminate sarcomas. Therefore, there is an immediate need to find novel sarcoma treatment strategies.

There are more than 160 kinds of chemical modification in RNA (Boccaletto et al., 2018; Mathlin et al., 2020). In both eukaryotes and prokaryotes, RNA methylation is a common post-transcriptional alteration (Courtney et al., 2019; Chen et al., 2021). N6-methyladenosine (m6A), C5-methylcytidine (m5C), N7-methylguanosine (m7G) and 2′-O-methylation were included in RNA methylation according to the different methylation sites (Zhang et al., 2021). RNA splicing (Zhao et al., 2014; Xiao et al., 2016), stability (Wang et al., 2014), translation (Meyer et al., 2015; Wang et al., 2015), DNA damage repair (Xiang et al., 2017) were regulated by RNA methylation, which then affected the occurrence and development of cancer (Li et al., 2017a; Zhang et al., 2017; Wang et al., 2019a). m7G is a kind of RNA methylation modification involving the addition of a methyl group to the seventh N position of RNA guanine (G) (Zhang et al., 2021). m7G modification is one of the most common forms of base modification in post-transcriptional regulation, which is broadly distributed in the 5'cap region of tRNA (Guy and Phizicky, 2014), rRNA (Sloan et al., 2017), siRNA (Pandolfini et al., 2019) and eukaryotic mRNA (Lin et al., 2018). The methyltransferase-like 1 (METTL1) and WD repeat domain 4 (WDR4) complex, which was a member of m7GRGs, primarily governed the processing, metabolism, and function of RNA (Alexandrov et al., 2002; Malbec et al., 2019). m7G methylation of tRNA and miRNA played a crucial role in the occurrence and development of cancers, such as liver cancer (Li et al., 2022), lung cancer (Ma et al., 2021), and colon cancer (Chen and Liu, 2021). However, few investigations on m7GRGs in sarcomas have been conducted.

The immune system has a considerable influence on sarcomas progression. Immune checkpoints and their ligands are expressed on the surface of various effector lymphocytes (Heinrich et al., 2018; Jiang et al., 2018). Sarcomas with a greater number of mutations are genetically heterogeneous and tend to have multiple neoantigens that can be targets for T cells and thus represent promising candidates for immune check-point inhibitors therapies. However, sarcomas with less mutated yet expresses immunogenic self-antigens (Nakata et al., 2021; Wang et al., 2021). Therefore, strategies to improve antigen presentation and T -cell infiltration may allow for successful immunotherapy. Therefore, the strategies of sarcoma and antigen presentation, T cell infiltration, and immune checkpoints may contribute to the success of immunotherapy.

A systematic bioinformatics investigation of m7GRGs in sarcomas was conducted in this study. Gene expression and mutation rates in sarcoma tissues were analyzed based on 27 m7GRGs. They were split into two subtypes by consensus clustering, and the signaling pathways of DEGs enrichment were analyzed. Prognostic m7GRGs in sarcomas were also analyzed using logrank test and univariate regression analysis. Cell experiment results revealed that prognostic m7GRGs are abundantly expressed in sarcoma cell lines. A prognostic model for predicting the overall survival (OS) and disease-specific survival (DSS) of sarcoma patients was created. There was a significant correlation between prognostic m7GRGs and immune cell infiltration, tumor mutation burden (TMB), microsatellite instability (MSI), and drug sensitivity. Moreover, there was a strong connection between m7GRGs and cuproptosis-related genes. Finally, ceRNA regulatory networks were constructed to screen the lncRNA-miRNA-mRNA networks that might affect the prognosis of patients with sarcoma. Our findings underscored the significance of m7GRGs in the formation of sarcomas, laying the groundwork for the use of m7G regulators in the treatment of sarcomas.

## Materials and methods

### Data sources and preprocessing

29 m7GRGs were identified based on published data in this study, including AGO2, CYFIP1, DCP2, DCPS, EIF3D, EIF4A1, EIF4E, EIF4E2, EIF4E3, EIF4G3, GEMIN5, IFIT5, LARP1, LSM1, METTL1, NCBP1, NCBP2, NCBP2L, NCBP3, NSUN2, NUDT10, NUDT11, NUDT16, NUDT3, NUDT4, SNUPN, and WDR4. In contrast, neither NUDT4B nor EIF4E1B was expressed in sarcomas within the TCGA dataset. Consequently, the remaining 27 m7GRGs were used for further analysis. Clinical information regarding sarcomas and m7GRGs expression was gathered from The Cancer Genome Atlas (TCGA) database for this investigation. (https://portal. gdc. cancer.gov//) (Tomczak et al., 2015). This study included 260 instances of sarcoma and two samples of non-cancerous tissue. The data utilized in this study were standardized data per million transcripts and their data distribution was close to the normal distribution, which was realized by R software (v4.0.3) “ggplot2”. Gene expression data were extracted to construct data matrices, which were then analyzed using wilcox test.

### Identification of molecular subgroups

Firstly, 27 m7GRGs were retrieved from the TCGA expression matrix. Consistency analysis was performed using the R software package Consensus Cluster Plus (v1.54.0), and the maximum number of clusters based on the consistent grouping of the twenty-seven genes was six (Wilkerson and Hayes, 2010). The cases of TCGA-SARCREAD disease were divided into two clusters based on the expression profile of m7GRGs. This procedure was repeated one hundred times to confirm the stability and reproducibility of the classification.

### Identification and functional enrichment analysis of DEGs

The DEGs between C1 and C2 subtypes were obtained by using the Limma package (version 3.40.2) in R software (Ritchie et al., 2015). The adjusted *p*-value was analyzed in the TCGA database to correct the false positive results. “Adjusted *p* < 0.05 and log2 (multiple changes) > 1.5 or log2 (multiple changes) <-1.5″was defined as the criteria for screening differential expression of mRNA. Gene MANIA (http://www.genemania.org) (Warde-Farley et al., 2010) is a software that elucidates the relationship between genes and data sets by building a network of gene interactions. In this study, Gene MANIA software was used to visualize the gene network of m7GRGs in terms of physical interaction, co-expression, prediction, co-mapping, and genetic interaction, as well as to assess its function. STRING database (https://string-db.org/) (version 11.0) (Szklarczyk et al., 2019) is a search tool for analyzing biological gene or protein interactions, including biological databases and networks for identified and predictable protein-protein interactions. Four differentially expressed m7GRGs were investigated using a PPI network to determine their interaction. The GO function and the enrichment of KEGG pathways were analyzed using “cluster Profiler” R packet (Yu et al., 2012). In addition, other potential biological pathways were identified using GSEA (http://software.broadinstitute.org/gsea/index.jsp) (Powers et al., 2018). According to TCGA data, DEGs were classified into upregulated and downregulated categories.10000 gene combinations were performed to identify pathways with significant changes in each analysis. The genes were regarded as enriched to meaningful pathways when p. adjust <0.05 and FDR (false discovery rate) < 0.25 (Ge et al., 2021). Statistical analysis and graphing were performed using the R package cluster profile (3.18.0).

### Immune infiltration, and immune checkpoint-related genes expression in two subgroups

The R software package immunedeconv (Sturm et al., 2020) was used for immune score assessment to compare the degree of immune cell infiltration in C1 (N = 156) and C2 (N = 104) subgroups by Wilcoxon test, by integrating six state-of-the-art algorithms, including TIMER, xCell, MCP-counter, CIBERSORT, EPIC, and quantTIseq. The expression of some immune checkpoint-related genes was also analyzed. The results were visualized through the R (v4.0.3) packages “ggplot2″ and “pheatmap.” The abundance of immunized cells infiltrated was analyzed through TIMER (https://cistrome.shinyapps. io/timer/) database (Li et al., 2017b) and TCGA database. In addition, the infiltration level of immune cell types was quantified by single sample GSEA (ssGSEA) in R packet “GSVA” (Hanzelmann et al., 2013).

### Expression of m7GRGs and survival analysis

The expression of m7GRGs in 260 sarcoma tissues and two paracancerous tissues was examined using the TCGA database. In addition, univariate Cox regression analysis was used to investigate the effect of m7GRGs on the prognosis of sarcomas. The logrank test and univariate Cox regression were used to derive Kaplan-Meier curves, *p* values, and hazard ratios (HR) with 95% confidence intervals (CI). Four m7GRGs (EIF4A1, EIF4G3, NCBP1, WDR4) with higher hazard ratios were screened from the Cox regression analysis plot. In addition, the relationship between the prognostic m7GRGs and the OS rate in sarcoma patients was analyzed, and the Area Under Curve under the receiver operator characteristic (ROC) curve was calculated.

### Cell lines and culture conditions

Cell lines such as 143B, SW982, SW872, osteoblast cell line (hFOB1.19, Punosai, Wuhan, China), synovial fibroblast (HFLS, Jennio Biotech, Guangzhou, China) and human preadipocyte line (HPA-v, sciencell) were used in this study. All cell lines were cultured in Dulbecco modified Eagle medium (DMEM; Gibco, Grand Island, NY, United States) supplemented containing 10% fetal bovine serum (Gibco, Grand Island, NY, United States), 100 U/ml penicillin and 100 U/ml streptomycin (Invitrogen, Carlsbad, CA, United States). The hFOB1.19 cell line was cultured in an incubator containing 5% CO_2_ at 34°C. The remaining cells were cultured in an incubator containing 5% CO_2_ at 37°C.

### RT-qPCR

Total RNA was extracted from cultured cells using high-purity RNA separation kits (Roche Diagnostics, Mannheim, Germany) and DNase I (Roche Diagnostics, Sigma-Aldrich) according to the manufacturer’s instructions. RNA was immediately reverse-transcribed using HiScript ®II 1st Strand cDNA Synthesis Kit (MR101-01 MR101 V azyme, Nanjing, China) according to the manufacturer’s instructions. Then, AceTaq ®qPCR SYBR Green Master Mix (Q121-03 azyme V azyme, China) was used for quantitative RT-PCR. The amplification conditions were: pre-denaturation at 95°C for 30 s, denaturation at 95°C for 5 s, annealing at 60°C for 30 s, and a total of 40 cycles according to 1 μ mol primer, 10 ng sample, 0.08 μ mol ROX dye, and 2 × SYBR Green Pro TaqHS Premix Ⅱ hybrid setting 20 μL Reaction system. Several specific primer sequences (Gene Pharma, China) were designed. The primer sequences are listed in Table 1. For PCR analysis, the mean cycle threshold (Ct) value of each target gene was standardized to that of the housekeeping gene GAPDH. The results were shown in a fold change using the 2^−ΔΔCT^ method.

**TABLE 1:** Primer sequences of genes.

Real-time quantitative PCR primer sequence	
Gene	Sequence (5′- 3′ on minus strand)
*GAPDH*	Fwd: GGA​GCG​AGA​TCC​CTC​CAA​AAT
	Rev: GGC​TGT​TGT​CAT​ACT​TCT​CAT​GG
*EIF4A1*	Fwd: ATG​GCA​CTA​GGA​GAC​TAC​ATG​G
	Rev: CCA​CGG​CTT​AAC​ATT​TCG​TCA
*EIF4G3*	Fwd: CCT​AGA​GCT​ACC​ATC​CCG​AAC
	Rev: GGG​CCA​CTA​TGA​CGG​TAC​TG
*NCBP1*	Fwd: GGA​GAG​CAA​CCT​AGA​AGG​CTT
	Rev: AGG​TAA​TAG​GCG​TGC​AAC​TGT
*WDR4*	Fwd: TAA​CCG​ATG​ACA​GTA​AGC​GTC​T
	Rev: TCT​CCT​CCG​AGG​CTA​TGA​AAG

### Construction and validation of the m7GRG prognostic model

A prognostic model was constructed using LASSO-Cox regression analysis based on the above prognostic m7GRGs. The prognostic m7GRG risk score was calculated as follows: Risk score = ∑I Coefficient (mRNAi) × Expression (mRNAi) according to the results of multivariate Cox regression analysis. Next, TCGA-SARC patients were divided into low-risk and high-risk subtypes according to the average risk score. The OS rates of the two subgroups were compared using Kaplan-Meier analysis, and time ROC research was done to estimate the accuracy of the model. The optimal truncated expression value is determined by the “surve_cutpoint” function of the “surviver” R package. According to the threshold value, the GSE17674, GSE71118, and GSE21050 data set patients downloaded from the GEO database are divided into high expression and low expression subgroups, further verifying the above results. The risk score of each included patient was calculated using the same model based on the characteristics of prognostic genes. Next, Kaplan-Meier and ROC curve were used to verify the predictive value of prognostic gene markers.

### Building a predictive nomogram

Each variable (including the *p*-value, and HR with 95% CI) was presented using univariate and multivariate cox regression analysis and forest plots by the “forest plot” package. The “rms” package was used to develop a Nomogram model for predicting 1, 3, and 5-year OS and DSS based on the results of multivariate cox proportional hazards analysis.

### Mutation analysis

CBioPortal (The cBio Cancer Genomics Portal) (http://www.cbioportal.org/index) provided a visual tool (Gao et al., 2013) for analyzing cancer gene data. Based on the TCGA database, cBioPortal was used to analyze the genomic map of m7GRGs to comprehend the mutation frequency in sarcomas.

### TMB, MSI, ESTIMATE score, and drug sensitivity

The relationship between Prognostic m7GRGs in sarcomas and TMB, MSI, and ESTIMATE score was analyzed using Spearman’s method. The chemotherapeutic response was predicted for each sample using the GDSC database (https://www.cancerrxgene.org/) (Yang et al., 2013). The half-maximum inhibitory concentration (IC50) of chemotherapeutic medicines was determined using ridge regression using the R package pRRophetic. Drug sensitivity and gene expression profiling data from cancer cell lines in the GDSC database were integrated in this study.

### Single cell analysis

The effect of prognostic m7GRGs on the expression of single cell subsets in the tumor microenvironment (TME) was investigated using TISCH (http://tisch.comp-genomics.org/) (Sun et al., 2021). TISCH is a scRNA-seq database that focuses on the TME and provides thorough annotation of single-cell cell types. Immune cells, stromal cells, and malignant cells were presented in this dataset. The t-distributed stochastic neighborhood embedding (t-SNE) map of SARC_GSE119352_mouse_ aPD1aCTLA4 and the heatmap of SARC_GSE119352_mouse_aPD1aCTLA4 were exhibited using the TISCH database to illustrate the effect of m7GRGs on the TME of sarcoma. The scatter diagrams of the correlation between immune infiltration levels between m7GRGs and tumor-associated fibroblasts (CAFs) were drawn by TIMER2.0 (http://timer.cistrome.org/) (Li et al., 2020).

### Cuproptosis-related gene expression analysis

The correlation between prognostic m7GRGs and cuproptosis-related gene expression in two hundred and sixty sarcoma samples was analyzed, and the difference in cuproptosis-related gene expression between the C1 group (N = 156) and C2 group (N = 104) was investigated. The genes FDX1, LIAS, LIPT1, DLD, DLAT, PDHA1, PDHB, MTF1, GLS, CDKN2A, SLC31A1, and ATP7B linked to cuproptosis were examined.

### Competing endogenous RNA network construction

Potential miRNA targets of prognostic CRGs were predicted using the ENCORI (http://starbase.sysu.edu.cn/) database (Li et al., 2014) and RNA22 (https://cm.jefferson.edu/rna22/interactive) database (Loher and Rigoutsos, 2012). The prognostic value of these putative miRNA targets in sarcomas was also confirmed using ENCORI, Kaplan-Meier Plotter, and TCGA-SARC cohort. The probable binding of lncRNAs to prognostic miRNAs was then predicted using the miRNet database (Chang et al., 2020) and ENCORI database. A miRNA-lncRNA regulatory network was established by Cytoscape (version 3.7.1; http://www.cytoscape.org/) software (Shannon et al., 2003). The prognostic significance of these possible lncRNA targets in sarcomas was investigated further. Finally, a lncRNA-miRNA-mRNA regulatory network was established.

## 3 Results

### RNA-seq transcriptional group data of m7GRGs in sarcomas

**FIGURE 1:**
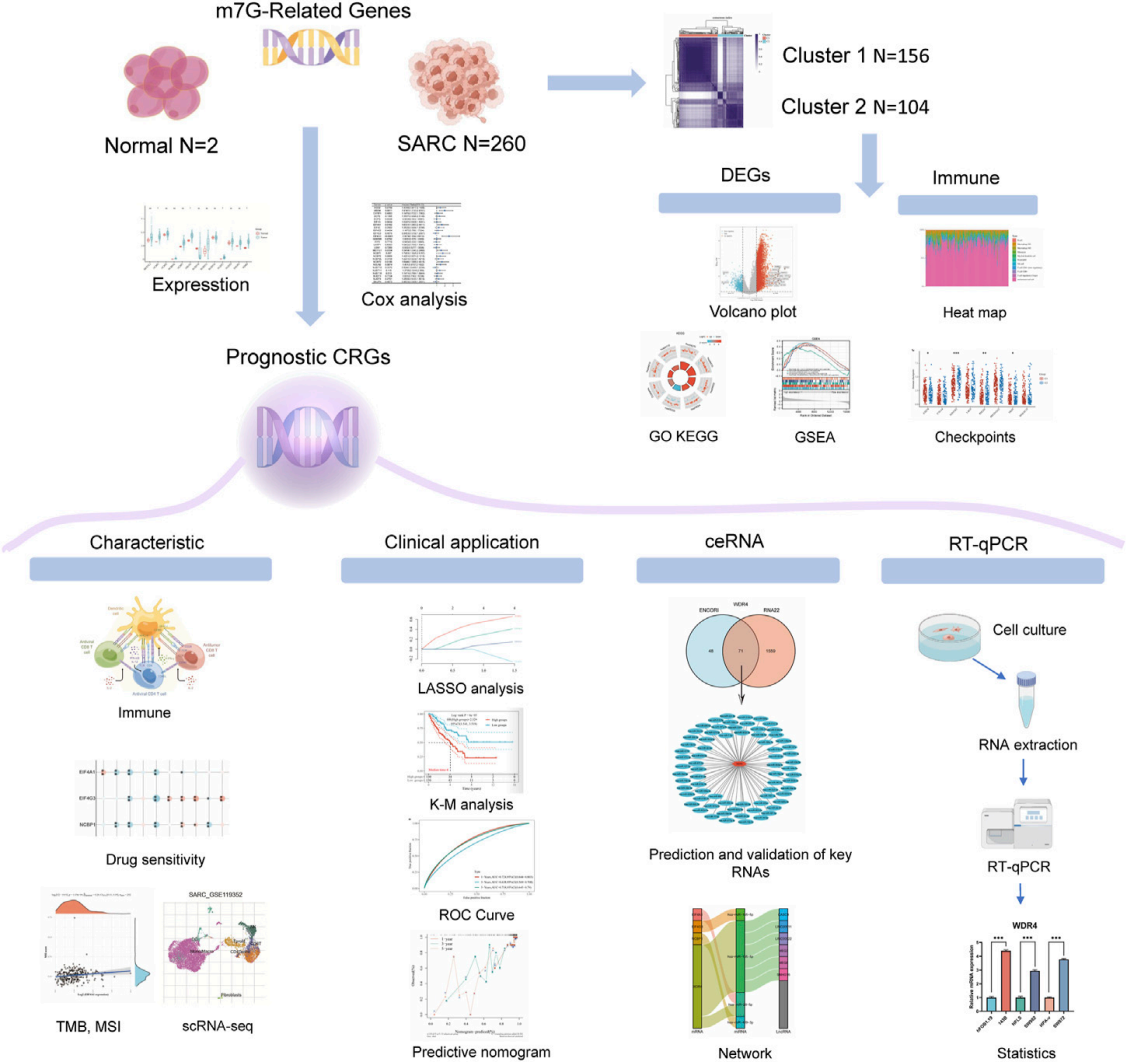
The flowchart of the present study.

**FIGURE 2:**
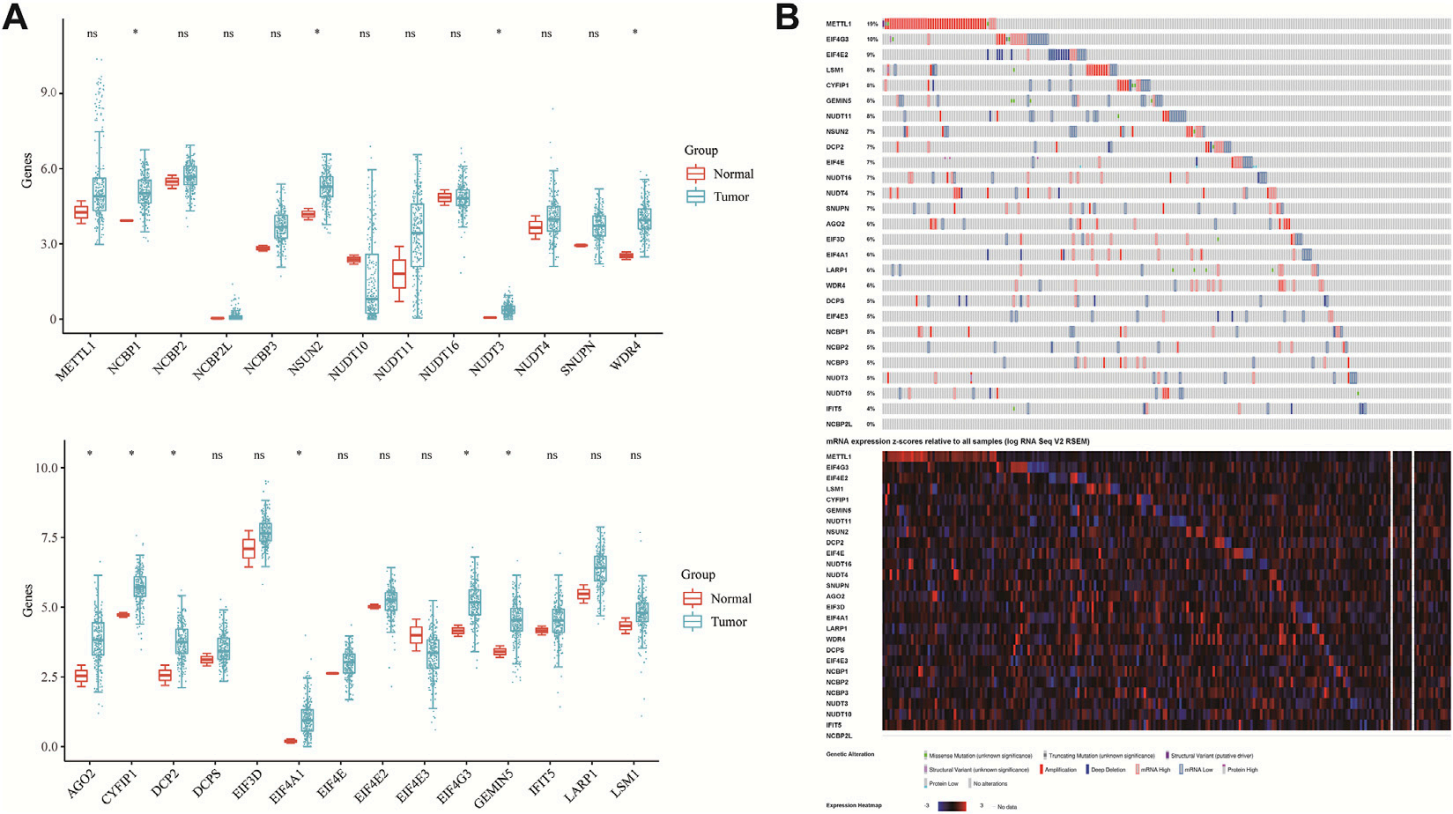
The variation and prognostic value of m7GRGs in sarcomas. **(A)** The expression of twenty-seven m7GRGs in sarcomas and paracancerous tissues. The upper and lower ends of the box represent the quartile range of values; the lines in the box represent the median; **(B)** the copy number of twenty-seven m7GRGs in the TCGA-SARC queue.

The flowchart of the study is illustrated in Figure 1. TCGA dataset was used to investigate the expression of twenty-seven m7GRGs in sarcomas and para-cancerous tissues. In cancer tissues, the expression of WDR4, NUDT3, NCBP1, DCP2, EIF4A1, NSUN2, CYFIP1, EIF4G3, GEMIN5, and AGO2 was upregulated compared with para-cancerous tissues (Figure 2A). In addition, the cBioPortal database was used to investigate the genetic variations of prognostic m7G in order to comprehend the etiology of m7GRG diseases. Eighty percent of the 255 sarcoma patients exhibited m7G regulatory gene alterations indicating a high frequency of somatic mutations in the m7GRGs of sarcomas. Figure 2B compares the mRNA expression z-scores of m7GRGs in sarcoma tissues with para-cancerous tissues. These findings demonstrated that m7GRGs are closely associated with sarcomas.

### Identification and analysis of m7GRG clusters in sarcomas

**FIGURE 3:**
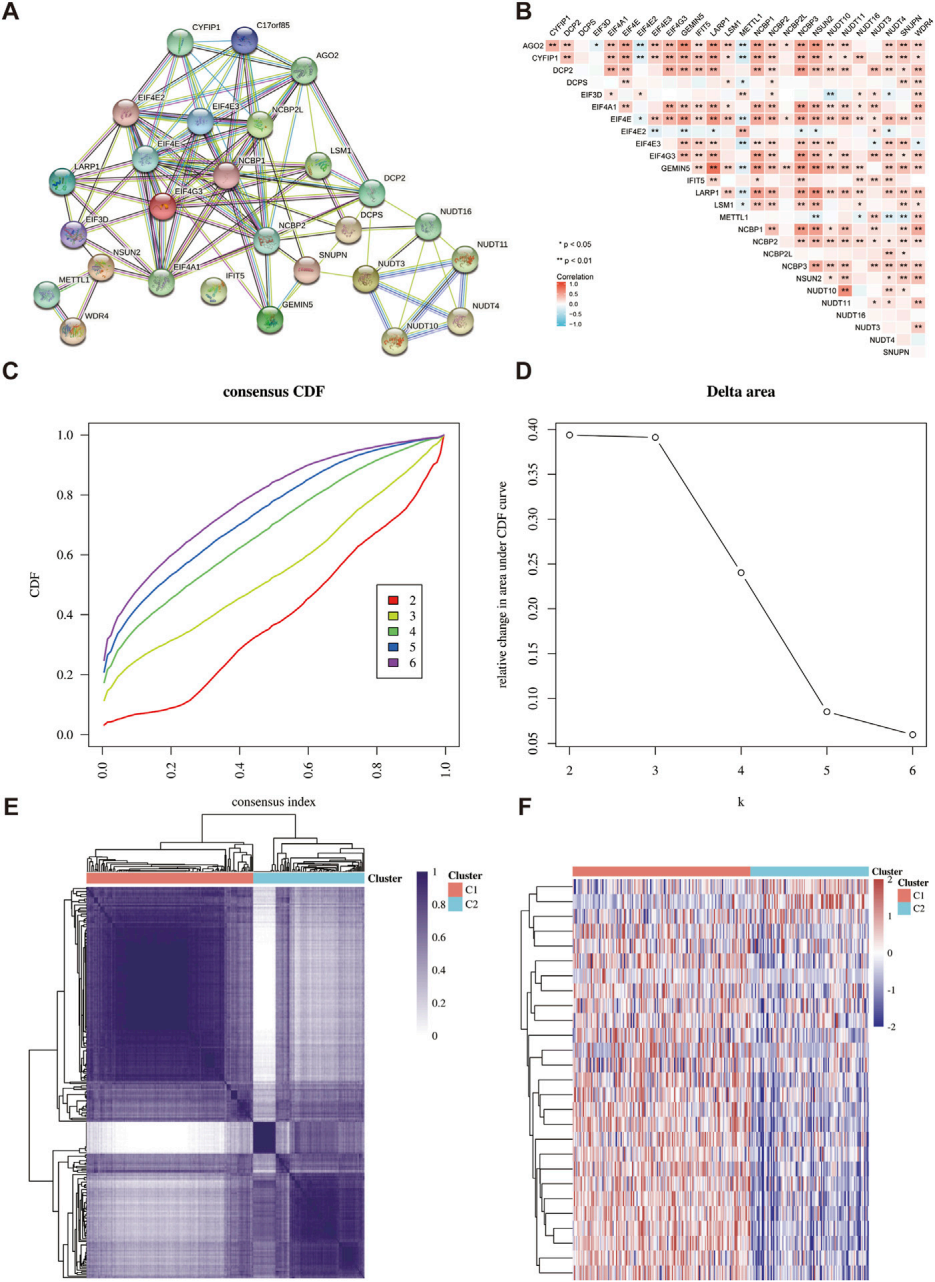
Identification of common clusters based on the expression of m7GRGs. **(A)** Protein-protein interaction of twenty-seven m7GRGs; **(B)** Pearson correlation analysis of twenty-seven m7GRGs expressions in sarcomas; **(C)** cumulative distribution function (CDF) (k = 2–6); **(D)** relative change of area under CDF curve (k = 2–6); **(E)** consensus clustering matrix (k = 2). **(F)** The heat map of m7GRG expression in different subgroups; red for high expression and blue for low expression.

The interaction between 27 m7GRGs was analyzed in order to comprehensively study the role of m7G modification in sarcomas. The protein-protein interaction network result showed a close relationship between m7G-related proteins (Figure 3A). In addition, Pearson correlation analysis was conducted to explore the correlation between the expression patterns of 27 m7GRGs in TCGA data sets. These results showed that most of the 27 m7GRGs were positively correlated (Figure 3B). Therefore, the biological function of these 27 m7GRGs and sarcomas were intimately related. 260 sarcoma samples were clustered in the TCGA database using consensus clustering to identify potential m7GRG clusters. All tumor samples were classified into k (k = 2–6) distinct clusters based on the expression of 27 m7GRGs in sarcomas. Subsequently, according to the cluster analysis findings, the number of clusters was set at two, suggesting that the sarcoma patients were properly split into two clusters (C1 and C2 clusters) (Figures 3C–F).

### DEGs and functional enrichment analysis

**FIGURE 4:**
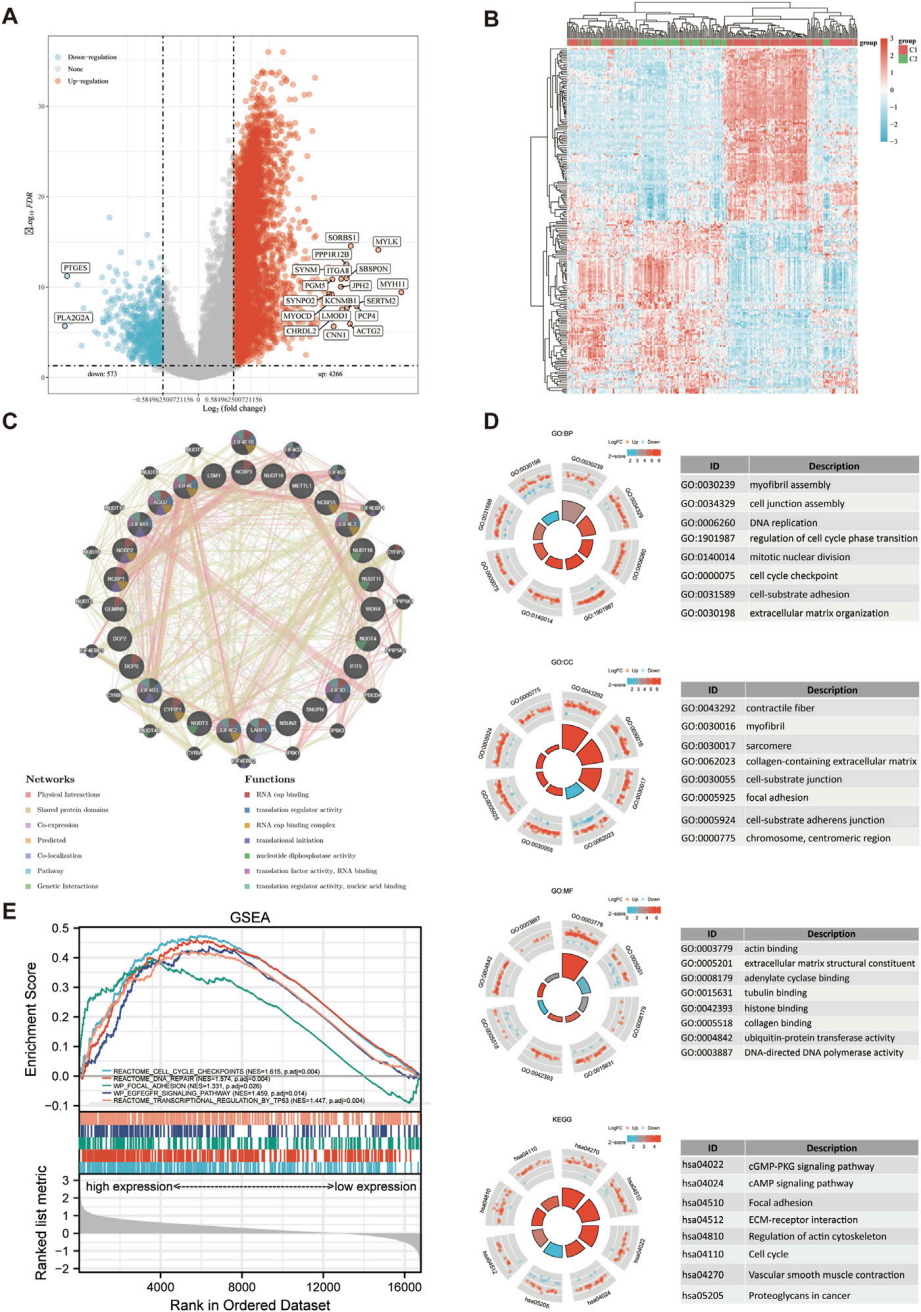
DEGs and functional enrichment analysis. **(A)** DEGs’ volcano plot between C1 and C2 subtypes. **(B)** DEGs’ heat map between C1 and C2 subtypes. **(C)** Gene Interaction Network. **(D)** Enriched item in GO analysis and KEGG analysis. **(E)** Enrichment plots from GSEA. BP, biological process; CC, cellular composition; MF, molecular function.

4,266 upregulated and 573 downregulated genes were included in the DEGs identified between C1 and C2 subtypes. Then, a volcano map (Figure 4A) and a heat map (Figure 4B) were constructed based on these DEGs. RNA cap binding, translation regulatory activity, translation initiation, nucleotide diphosphatase activity, translation factor activity, RNA binding, and nucleic acid binding were among the processes associated with the functions of the significantly co-expressed genes in this network, as determined by GeneMANIA. (Figure 4C). The identified up-and-down-regulated DEGs were further subjected to GO and KEGG enrichment analysis. Biological process (BP) analysis showed that the DEGs were mainly concentrated in myofibril assembly, cell junction assembly, DNA replication, cell cycle phase transition regulation, and extracellular matrix tissue. The Cell Composition (CC) analysis revealed that the majority of DEGs were found in contractile fibers, myofibril, sarcomere, extracellular matrix containing collagen, cell-matrix junction, adhesion plaque. Molecular Function (MF) analysis showed that the DEGs were mainly concentrated in actin binding, extracellular matrix structural components, adenylate cyclase binding, tubulin binding, and collagen binding. The DEGs were most abundant in adhesion plaque, cGMP-PKG signal route, cAMP signal pathway, ECM-receptor interaction, and cell cycle, as determined by KEGG analysis (Figure 4D; Supplementary Table S1). The results of Gene Set Enrichment Analysis (GESA) showed that m7GRGs were tightly associated with adhesion plaque, cell cycle checkpoint, DNA repair, TP53 transcriptional regulation, and EGFEGFR signal pathway. Due to the activation of these pathways, the likelihood of tumor development and progression was raised (Figure 4E; Supplementary Table S2).

### Analysis of the correlation between immune infiltration and immune checkpoints

**FIGURE 5:**
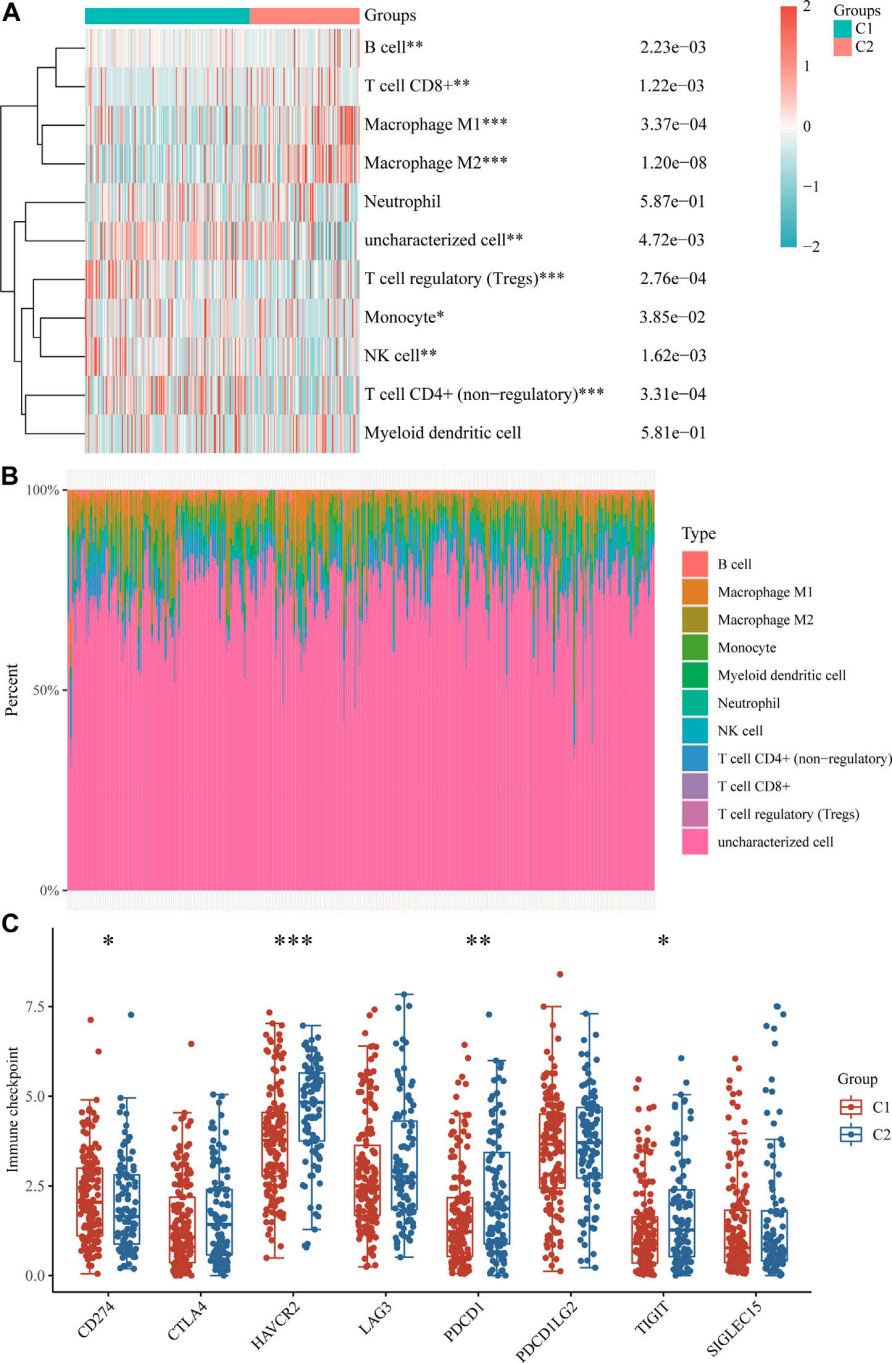
Immune infiltration estimated by QUANTISEQ algorithm and the expression distributions of eight immune checkpoint-related genes in sarcoma subgroups. **(A)** Immune cell score heat map. **(B)** Proportions of eleven types of immune cells shown for each sarcomatous patient by a histogram. **(C)** The expression distributions of eight immune checkpoint-related genes in sarcomatous subgroups. *p < 0.05; **p < 0.01; ***p < 0.001.

The results showed that the expression of m7GRGs was closely related to tumor-infiltrating immune cells, which included CD4^+^ T cells, CD8^+^ T cells, B cells, NK cells, macrophages, myeloid dendritic cells, monocytes, endothelial cells, and neutrophils (Figures 5A, B; Supplementary Figures S1A–E). Finally, the results of immune checkpoints’ expression showed significant differences in CD274, HAVCR2, PDCD1, and TIGIT between the two subtypes (Figure 5C), and m7GRGs might be a predictive marker for the treatment of sarcomas by targeting immune checkpoints.

### DEGs clinicopathological characteristics and prognostic models

**FIGURE 6:**
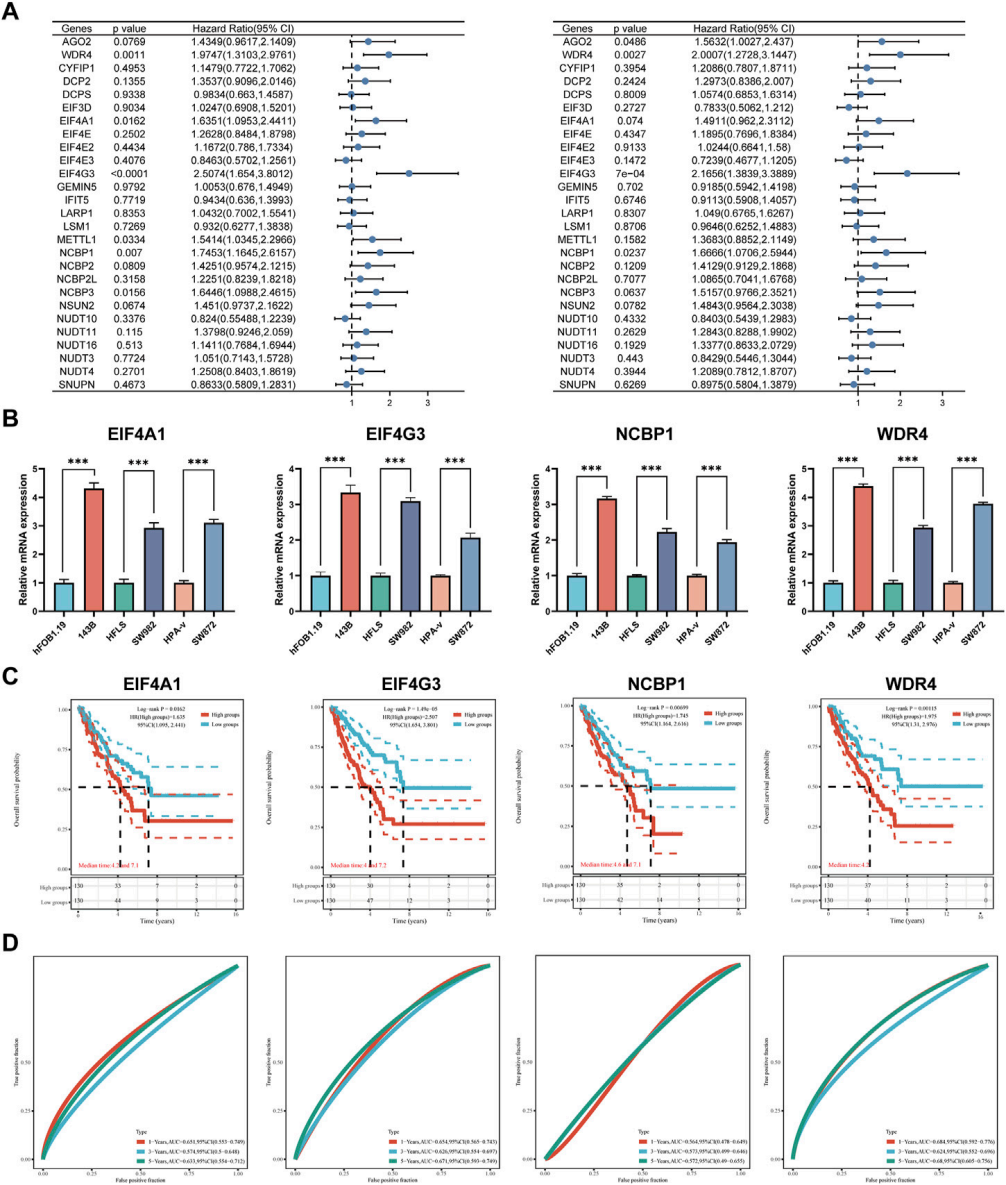
DEGs and prognostic models. **(A)** Analysis of four prognostic m7GRGs from univariate Cox regression analysis plots; **(B)** mRNA expression of prognostic m7GRGs in sarcoma cell lines and the corresponding normal cell lines; **(C, D)** Prognostic value of four m7GRGs (EIF4A1, EIF4G3, NCBP1, and WDR4) in sarcoma patients (OS curve of high/low expression group).

Clinical characteristics of patients with sarcoma from TCGA cohort (Supplementary Table S3). To determine the clinical importance of m7GRGs in sarcoma tissues, the connection between the expression of C1 and C2 subtypes and various clinicopathological characteristics was examined using the TCGA database. The research revealed substantial differences between group C1 and group C2 in gender, race, and new tumor type (Supplementary Figure S3). Univariate Cox regression analysis was performed to identify prognostic m7GRGs. Survival analysis suggested that EIF4A1, EIF4G3, METTL1, NCBP1, NCBP3, and WDR4 were potential risk factors for OS (Figure 6A). Moreover, AGO2, EIF4G3, NCBP1, and WDR4 were potential risk factors for DSS in sarcomas. The mRNA expression of EIF4A1, EIF4G3, NCBP1, and WDR4 in sarcomatous cell lines (143B, SW982, and SW872) was considerably upregulated compared to their equivalent normal cell lines (Figure 6B). Based on the above prognostic analysis, Kaplan-Meier survival curve revealed that the OS rate of sarcoma patients with high expression of EIF4A1 (HR = 1.64, *p* = 0.016), EIF4G3 (HR = 2.52, *p* = 0), NCBP1 (HR = 1.74, *p* = 0.007) and WDR4 (HR = 1.98, *p* = 0.001) was lower. (Figures 6C, D). Consequently, four genes with prognostic values (EIF4A1, EIF4G3, NCBP1, and WDR4) were identified.

### Construction and validation of the m7GRG prognostic model by TCGA dataset and external databases

**FIGURE 7:**
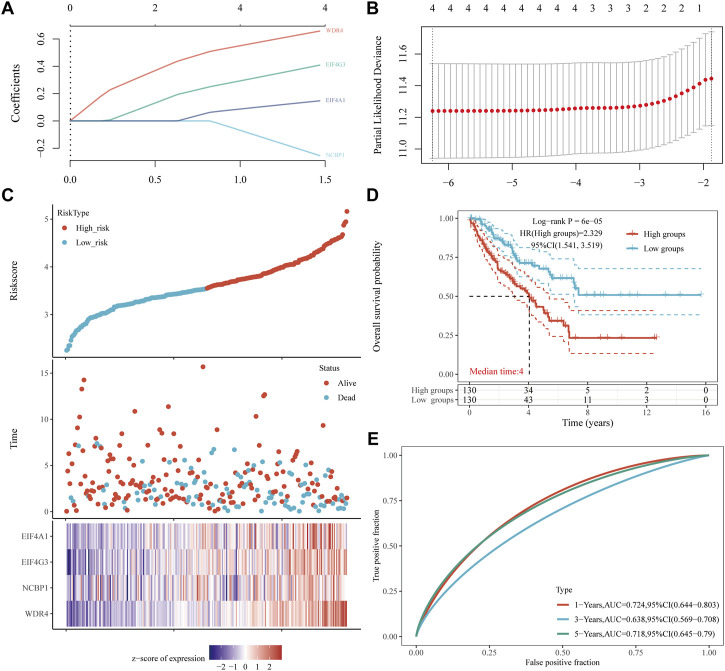
Construction of a prognostic m7GRG model. **(A)** LASSO coefficient profiles of prognostic m7GRGs, **(B)** Plots of the ten-fold cross-validation error rates. **(C)** Distribution of the risk score, survival status, and the expression of prognostic m7GRGs in sarcomas. **(D, E)** OS curves of sarcoma patients in the high-/low-risk group and the ROC curve for measuring the predictive value.

A prognostic gene model was constructed using LASSO Cox regression analysis based on prognostic m7GRGs (Figure 7; Supplementary Figures S3A, B). Risk score = (0.1472) *EIF4A1 + (0.4087)*EIF4G3 (−0.2538)*NCBP1 + (0.6578)*WDR4 was applied to the calculation of OS in sarcoma patients. Sarcoma patients were divided into two groups according to the risk scores, survival status, and the expression of EIF4A1, EIF4G3, NCBP1, and WDR4 (Figures 7C, D). The risk of mortality rose and survival duration reduced with the rise of risk score (Figure 7C). Sarcoma patients with high-risk scores had a decreased likelihood of OS (median time = 4 years, p = 6e-5) according the result of the Kaplan-Meier curve (Figure 7D). The area under the ROC curve (AUC) for the 1-year, 3-year and 5-year ROC curves were 0.724, 0.638, and 0.718 respectively (Figure 7E). The same analysis was carried out on the analysis of DSS. Sarcoma patients were divided into two groups based on the distribution of risk scores, survival status, and expression of EIF4G3 and WDR4 (Supplementary Figures S3C, D) and the risk score = (0.1036)*EIF4G3 + (0.5255)*WDR4 was applied to the calculation of DSS. The DSS is shorter the higher the patient’s risk score (HR = 1.974 95% CI = 1.259–3.095, log-rank *p* = 0.00302) (Supplementary Figures S3D). The area under the ROC curve (AUC) in the 1-year, 3-year, and 5-year ROC curves was 0.696, 0.621, and 0.693, respectively (Supplementary Figure S3E). The prognostic m7GRG model showed a substantial correlation between sarcoma patient survival rate and m7GRGs. In order to verify the predictive value of the four gene characteristics, we calculated the patient’s risk score GEO data set (GSE17674, GSE71118 and GSE21050) using the same formula, which was consistent with the results of the TCGA cohort. Distribution of risk score, survival time and m7GRGs expression in each SARC patient (Supplementary Figures S4A, S5A, S6A). The OS of patients in the high-risk group was significantly lower than those in the low-risk group (*p* = 0.00137, *p* = 0.000474 and *p* = 0.00107) (Supplementary Figures S4B, S5B, S6B). The AUC of 1-year, 3-year and 5-year OS is 0.703, 0.637 and 0.709 respectively (Supplementary Figutre S4C), 0.521, 0.55, and 0.528 respectively (Supplementary Figure S5C) and 0.509, 0.54 and 0.53 respectively (Supplementary Figure S6C). To sum up, these results confirm the effectiveness of our risk scoring model. The four gene characteristics can predict OS in SARC.

### Building of a predictive nomogram

**FIGURE 8:**
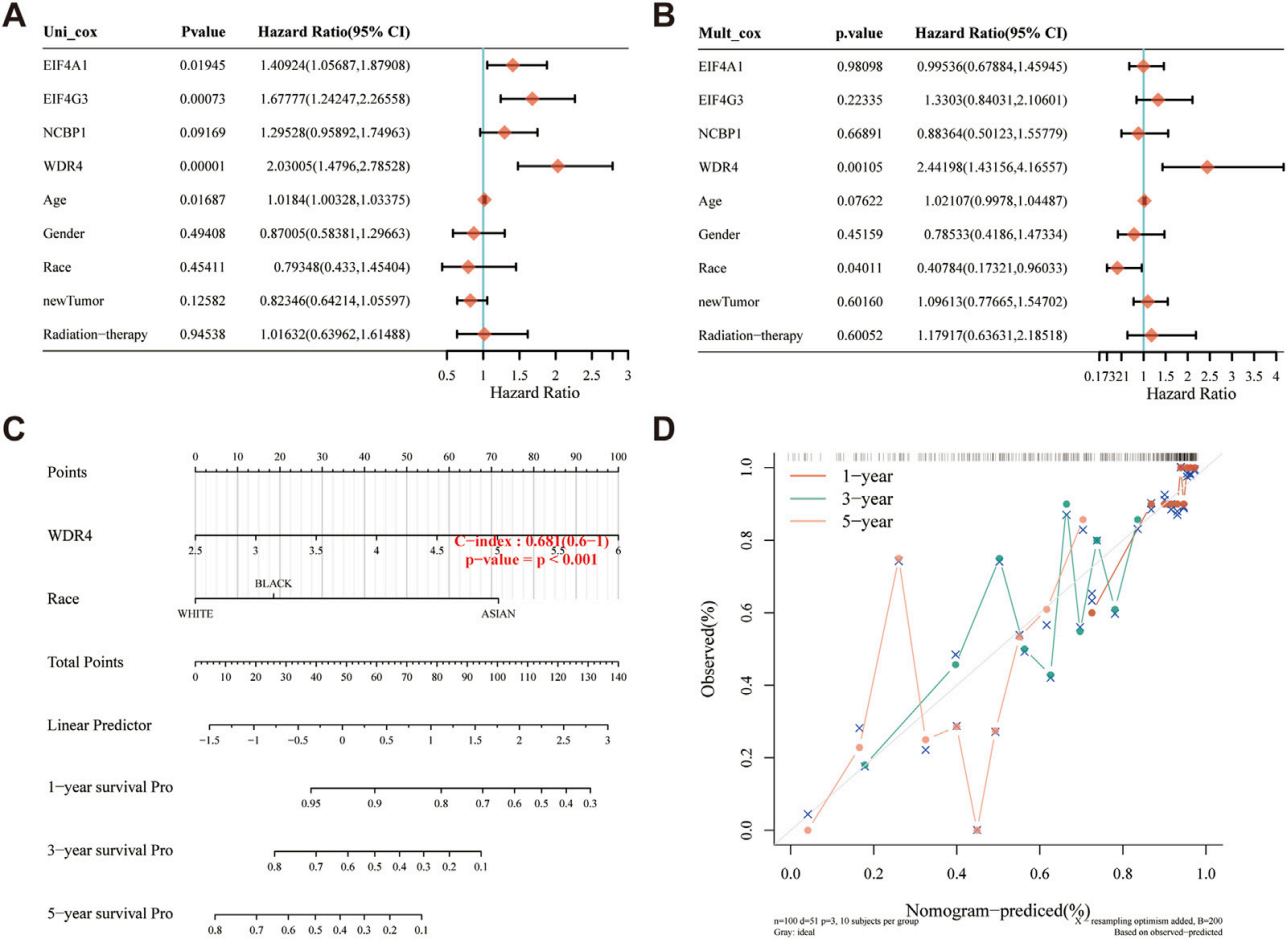
Construction of a predictive nomogram. **(A, B)** Hazard ratios and *p*-value of the constituents involved in univariate and multivariate Cox regression analysis considering the clinical information and prognostic m7GRGs in sarcomas. **(C)** Nomogram to predict the 1-year, 3-year and 5-year OS rate of SARC patients. **(D)** Calibration curve for the OS nomogram model in the discovery group. The dashed diagonal line represents the ideal nomogram.

A predictive nomogram was built to predict the survival probability of sarcoma patients. The results of the univariate and multivariate analyses revealed that WDR4 expression and race were independent factors affecting the prognosis of sarcoma patients (Figures 8A, B; Supplementary Figures S7A, B). The predictive nomogram suggested that the 3-year and 5-year OS rates and DSS rates were accurately predicted compared with an ideal model in the entire cohort (Figures 8C, D; Supplementary Figures S7C, D).

### Prognostic m7GRGs interfere with immune cell infiltration in sarcomas

**FIGURE 9:**
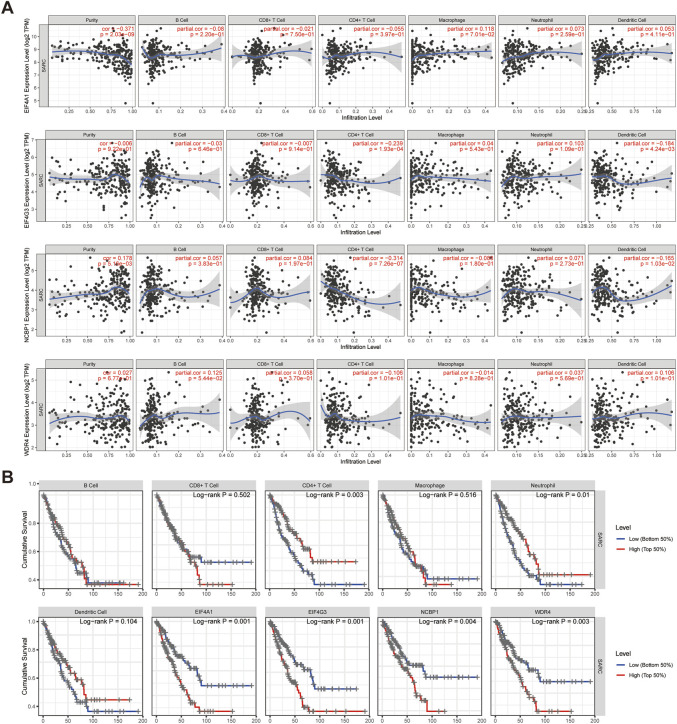
Prognostic m7GRGs intervene in immune infiltration in sarcomas. **(A)** Correlation between the abundance of immune cells and the expression of prognostic m7GRGs in sarcomas; **(B)** The relationship between the expression of EIF4A1, EIF4G3, NCBP1, WDR4, B cells, CD8^+^ T cells, CD4^+^ T cells, macrophages, neutrophils, dendritic cells and the cumulative survival rate in sarcomas.

The correlation between the expression of prognostic m7GRGs (EIF4A1, EIF4G3, NCBP1, WDR4) and immune infiltration in sarcomas was investigated using the TIMER database and TCGA database. According to TIMER data, EIF4G3, and NCBP1 were negatively connected with CD4^+^ T cells and dendritic cells, however, EIF4A1 and WDR4 were not substantially correlated with immune cell infiltration in sarcomas (Figure 9A). Higher amounts of CD4^+^ T cells and neutrophils were associated with a better prognosis, as determined by immune survival testing. In addition, the prognosis was worse the greater the expression of EIF4G3 and NCBP1 (Figure 9B). Then, the infiltration of twenty-four immune cell types in sarcomas was determined using ssGSEA method, and the relationship between prognostic m7GRGs and immune cell infiltration was studied by Spearman analysis. The results showed that the high expression levels of prognostic m7GRGs were significantly negatively correlated with most immune cells. EIF4G3 and NCBP1 were positively correlated with T helper cells, Th2 cells, but negatively correlated with pDC, Cytotoxic cells, DC, T cells. In addition, EIF4A1 and WDR4 were positively correlated with T helper cells, and Th2 cells and negatively correlated with Mast cells, pDC, DC, NK cells, and Cytotoxic cells (Supplementary Figure S8A). Furthermore, EIF4G3, NCBP1, and WDR4 were negatively correlated with the ESTIMATE score (Supplementary Figure S8B). The findings demonstrated a correlation between m7GRGs and immune infiltration of tumors.

### TMB, MSI, and drug sensitivity analysis

**FIGURE 10:**
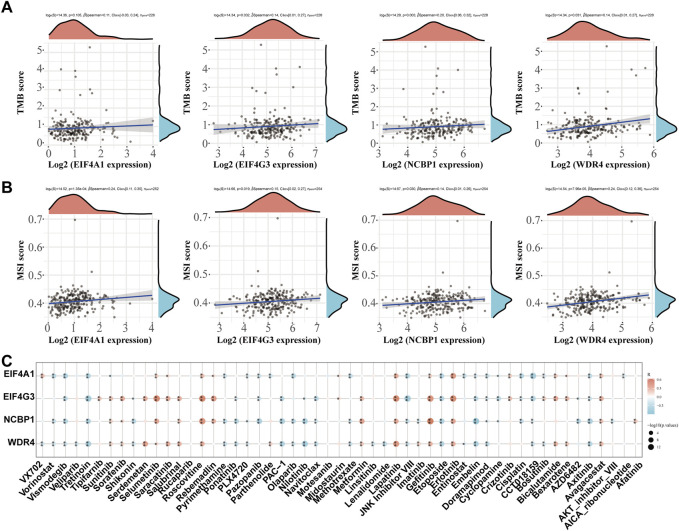
TMB, MSI and drug sensitivity. **(A)** Correlation between the expression of prognostic m7GRGs and TMB in sarcomas; **(B)** Correlation between prognostic m7GRGs and TMB in sarcomas; **(C)** Correlation between prognostic m7GRGs and antitumor drugs in sarcomas.

The correlation between prognostic m7GRGs and TMB, MSI was analyzed in sarcomas to determine whether m7GRGs can be used as biomarkers for screening chemotherapeutic medications. MSI and TMB may be utilized as predictors of the immunotherapy response of certain cancers (Rizzo et al., 2021). The results showed that EIF4G3 (*p* = 0.032), NCBP1 (*p* = 0.003), and WDR4 (*p* = 0.031) were positively correlated with TMB (Figure 10A). EIF4A1 (*p* = 1.35e−04), EIF4G3 (*p* = 0.019), NCBP1 (*p* = 0.030), and WDR4 (*p* = 7.96e−05) were strongly positively associated with MSI (Figure 10B). Finally, the gene expression patterns of cancer cell lines from the Genomics of Drug Sensitivity in Cancer database were combined in order to comprehensively investigate the potential therapeutic benefit of the EIF4A1, EIF4G4, NCBP1, and WDR4 genes. The result of Pearson correlation analysis showed that the expression of EIF4A1, EIF4G3, NCBP1 and WDR4 was favorably correlated with Selumetinib, Roscovitine, Lapatinib, Gefitinib, Erlotinib and Avagacestat, but negatively correlated with Vismodegib, Tretinoin, JNK inhibitor VIII, Etoposide, Embelin, Doramapimod, CCT018159 and Axitinib (Figure 10C).

### Single-cell RNA data analysis

**FIGURE 11:**
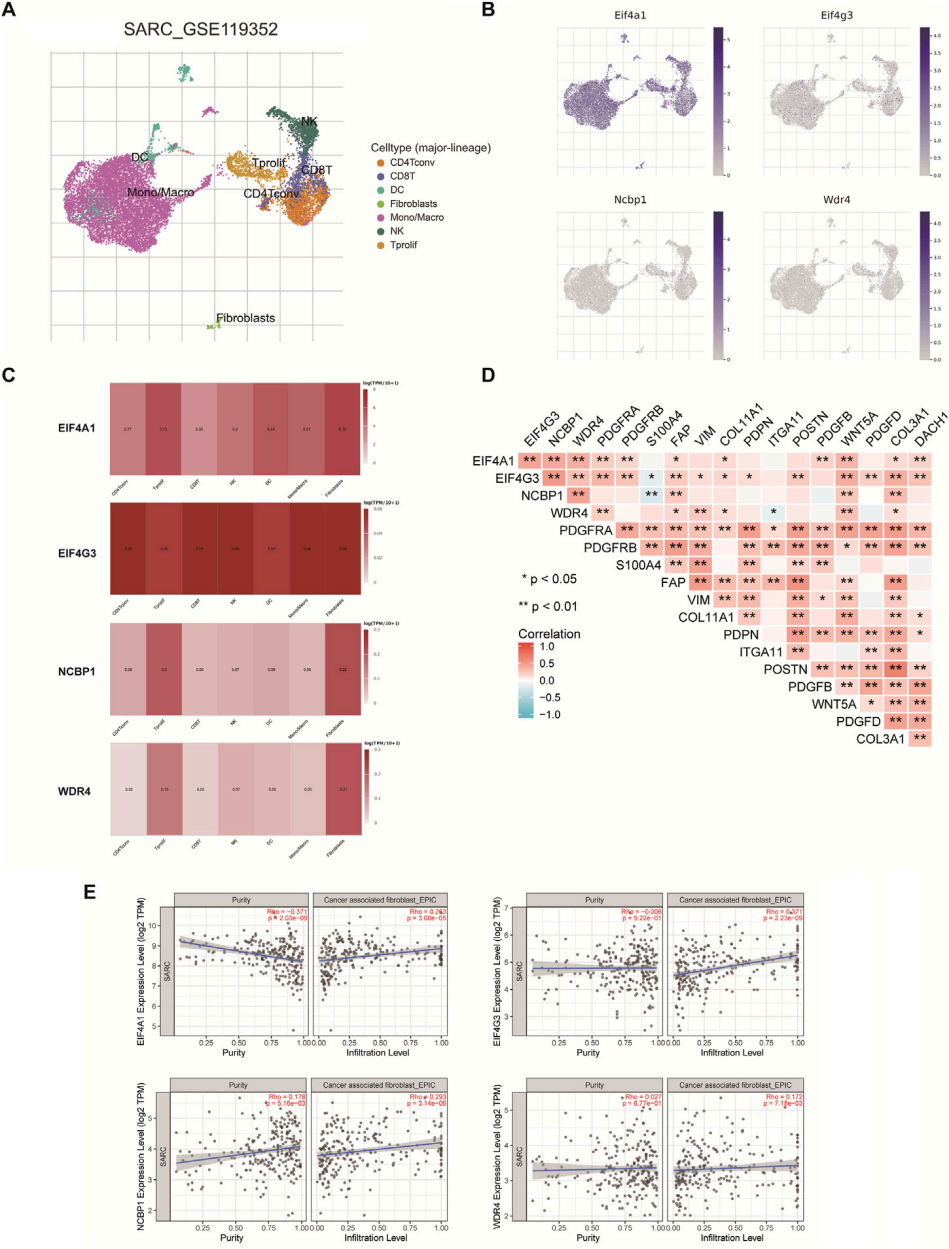
The expression of m7GRGs in different immune cell types in sarcomas. **(A)** Cluster diagram of cell types in scRNA seq data. t-SNE diagram showing the expression of different immune cells (SARC_GSE119352_mouse_APD1aCTLA4) in sarcoma tissues; **(B, C)** Characteristic maps of prognostic m7GRGs obtained from scRNA seq data; **(D)** Correlation between m7GRGs and CAF-related markers; **(E)** Correlation between prognostic m7GRGs and CAFs infiltration; Correlation analysis was completed by TIMER2.0; **p < 0.01; Cancer associated fibroblasts (CAFs).

Extracellular matrix (ECM), CAF, muscle fibroblasts, and immune cells comprise the majority of TME. CD4Tconv, Tprolif, CD8T, NK, DC, Mono/Macro, Fibroblasts were annotated by single-cell RNA sequencing analysis (Figure 11A). The results showed that EIF4A1, EIF4G3, NCBP1, and WDR4 were considerably expressed in fibroblasts and were strongly connected with immune cells, stromal cells, and malignant cells (Figures 11B, C). Consequently, the relationship between m7GRGs expression and CAFs related biomarkers was explored further. The correlation between m7GRGs and CAF markers such as PDGFRA, PDGFRB, and S100A4 was extensive (as shown in Figure 11D). Meanwhile, immune infiltration analysis was used to determine the correlation between the prognostic m7GRGs and CAFs infiltration. The results showed that EIF4A1 (n = 260, Rho = 0.263, *p* = 3.08e−05), EIF4G3 (n = 260, Rho = 0.371, *p* = 2.23e−09), NCBP1 (n = 260, Rho = 0.293, *p* = 3.14e−06) and WDR4 (n = 260, Rho = 0.172, *p* = 7.18e−03) were positively correlated with CAFs infiltration (Figure 11E).

### Correlation between m7GRGs and cuproptosis-related genes in sarcomas

**FIGURE 12:**
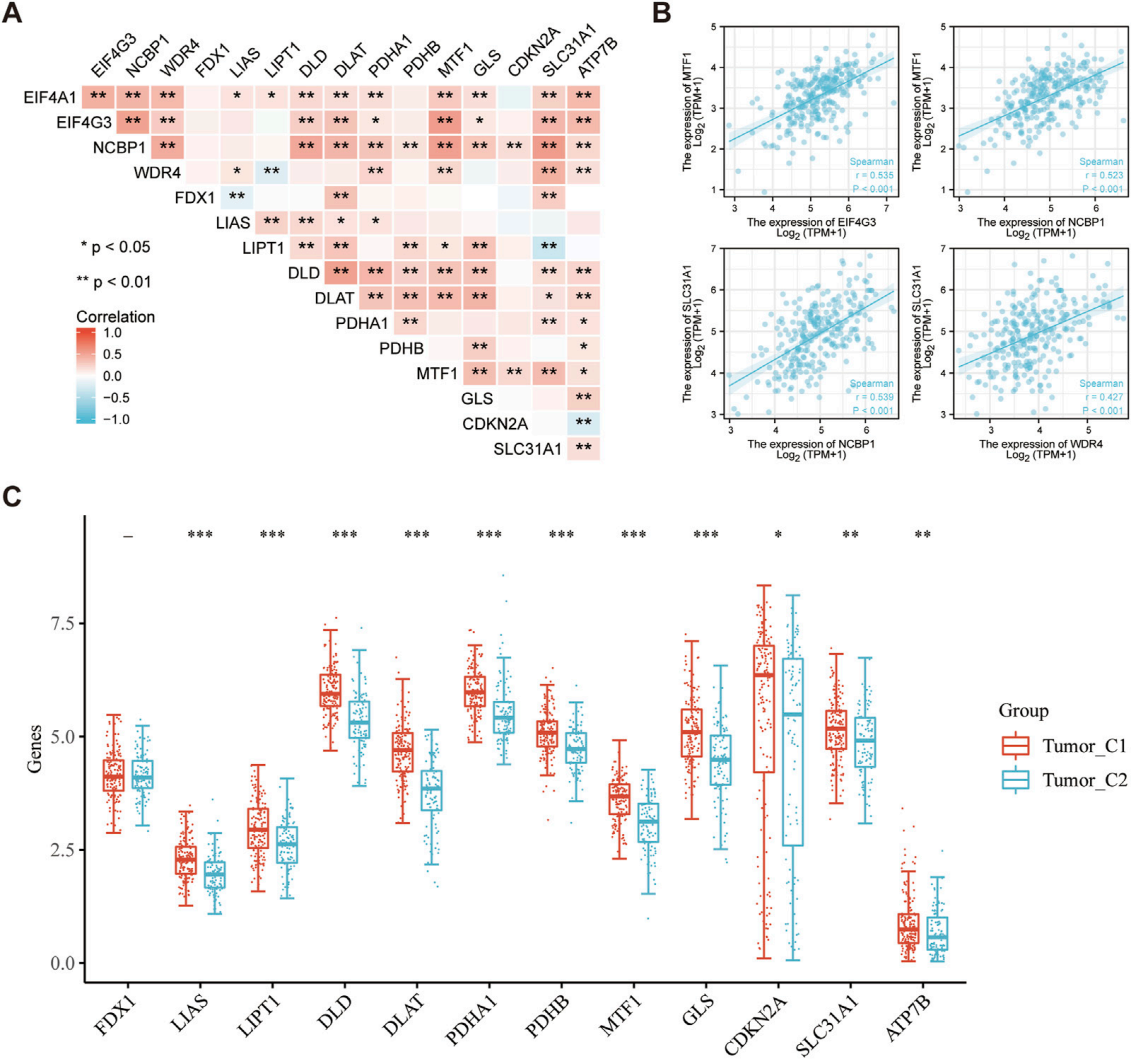
Significant correlation between m7GRGs and cuproptosis-related genes. **(A)** Heat map showing correlation between m7GRGs and cuproptosis-related genes; **(B)** Scatter plot of correlation between prognotic m7GRGs and cuproptosis-related genes; **(C)** Differential expression of cuproptosis-related genes between C1 and C2 subtypes.

Cuproptosis is a novel method of programmed cell death in which copper may directly join with fatty acylation components in the tricarboxylic acid cycle (Tsvetkov et al., 2022). Cuproptosis is intimately associated with the progression of cancers such as kidney cancer, liver cancer, bladder cancer, etcetera (Zhang et al., 2022a; Bian et al., 2022; Song et al., 2022). The correlation between m7GRGs and cuproptosis-related genes was analyzed using the Spearman correlation coefficient in order to explore the novel mechanism of the occurrence and development of sarcomas. The result demonstrated that prognostic m7GRGs were tightly associated with cuproptosis-related genes (Figure 12A). EIF4G3 and MTF1 exhibited a substantial positive connection (R = 0.535; *p* < 0.01), as did NCBP1 and MTF1 (R = 0.524; *p* < 0.01), NCBP1 and SLC31A1 (R = 0.539; *p* < 0.01), and WDR4 and SLC31A1 (R = 0.427; *p* < 0.01). A scatter plot of the correlation between prognostic m7GRGs and cuproptosis-related genes was created based on these results (Figure 12B). In addition, most cuproptosis-related genes were differentially expressed in the two sarcoma subtypes (Figure 12C). The association between m7GRGs and cuproptosis-related genes was validated by the aforementioned findings.

### Prediction and validation of upstream key miRNAs

The intersection of ENCOR1 and the RNA22 database yielded 50 pairs of EIF4A1-miRNAs, 83 pairs of EIF4G3-miRNAs, 47 pairs of NCBP1-miRNAs, and 71 pairs of WDR4-miRNAs. Using Cytoscape, a possible miRNAs gene network was created. (Supplementary Figure S9A). A negative correlation between the predicted mRNA and the predicted miRNA was expected according to traditional mechanism of miRNA in negative regulation of gene expression. One pair of miRNA-EIF4A1, 1 pair of miRNA-EIF4G3, 1 pair of miRNA-NCBP1 and two pairs of miRNAs-WDR4 were substantially negatively correlated among these miRNA-mRNA interactions (Supplementary Figure S9B). Theoretically, miRNAs that bind to high expression EIF4A1, EIF4G3, NCBP1, and WDR4 should be down regulated in sarcomas and show poor prognosis. The prognostic role and expression of these potential miRNAs in sarcomas were further verified using the ENCOR1 database. The findings revealed that only the low expression of hsa-miR-195-5p had a substantial unfavorable prognosis (Supplementary Figure S9C). WDR4-hsa-miR-195-5p might represent a critical pathway that mediated the incidence and development of sarcomas, incorporating the findings of correlation and survival study.

### Prediction and validation of key lncRNAs binding to potential miRNAs

**FIGURE 13:**
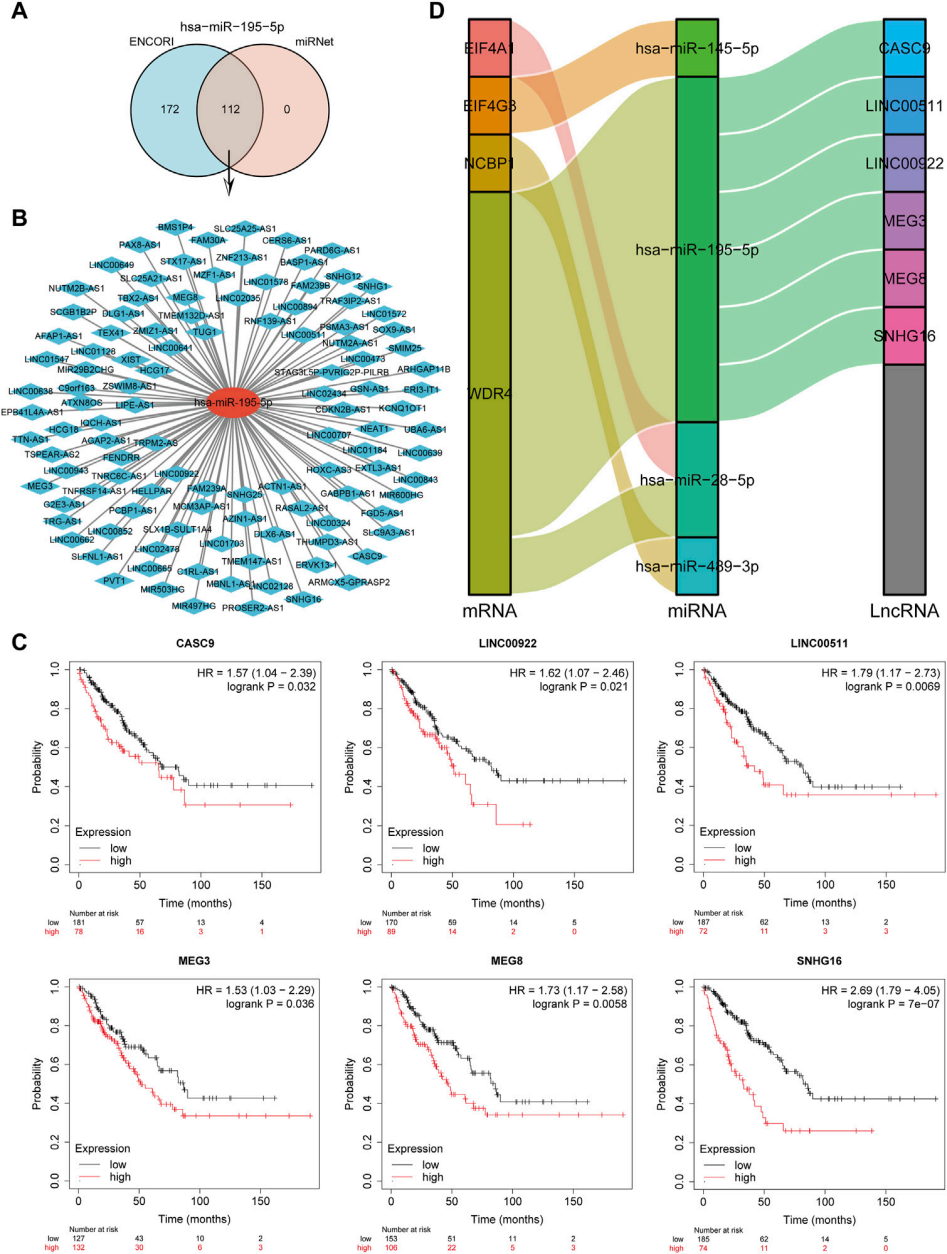
Screening of the regulatory axis of lncRNA-miRNA-CRGs in sarcomas. **(A)** Potential lncRNAs associated with hsa-miR-195-5p predicted by ENCORI and miRNet databases. **(B)** Potential miRNA-lncRNA network constructed using Cytoscape software. **(C)** Expression and prognostic value of six potential lncRNAs in sarcomas. **(D)** LncRNA-miRNA-mRNA triple regulatory network affecting the prognosis of sarcomas.

LncRNAs bind to miRNAs, which provide a biological purpose by modulating the expression of target genes. The lncRNAs potentially binding to hsa-miR-195-5p were predicted by the intersection of ENCORI and miRNet databases, yielding a total of 121 lncRNAs targeting hsa-miR-195-5p (Figure 13A). A miRNA-lncRNA regulatory network was established using Cytoscape software for better visualization (Figure 13B). According to the ceRNA hypothesis (Salmena et al., 2011), lncRNAs enhance mRNA expression by binding competitively to miRNAs. Consequently, lncRNAs were negatively correlated with miRNAs or positively correlated with mRNAs. The ENCORI database identified the association between lncRNAs and hsa-miR-195-5p, and the findings indicated that 10 lncRNAs were substantially associated with hsa-miR-195-5p and WDR4 (Supplementary Table S4). Subsequently, the prognostic value of lncRNAs in sarcomas was assessed by the Kaplan-Meier plotter. CASC9, LINC00922, LINC00511, MEG3, MEG8, and SNHG16 were significantly related to the poor prognosis of sarcoma patients (Figure 13C). Finally, a critical mRNA-miRNA-lncRNA regulation network related to the prognosis of sarcoma patients was identified (Figure 13D).

## 4 Discussion

RNA methylation is a prevalent method of RNA modification in prokaryotic and eukaryotic cells. They may be divided into m6A, m5C, and m7C according to distinct locations of methylation modification (Zhang et al., 2021). m7G is the modification of RNA guanine (G) by adding methyl group at the 7th N position (Zhang et al., 2021), mostly in tRNA, rRNA, and mRNA 5’cap, which plays a crucial role in maintaining RNA processing, metabolism, stability, protein translation (Ramanathan et al., 2016). Through m7G alteration of tRNA or miRNA, the METTL1/WDR4 complex influences the course of several malignancies, such as liver cancer (Li et al., 2022), lung cancer (Ma et al., 2021), and colon cancer (Chen and Liu, 2021). However, the role of m7G methylation modification in sarcomas remains unclear. To guide future investigation of m7GRGs in sarcomas, a bioinformatics analysis was performed on publicly available sequencing data, and RT-qPCR was employed for experimental validation.

Firstly, 27 m7GRGs were retrieved from the TCGA expression matrix and separated into subgroups C1 and C2. m7G regulatory genes showed high expression mainly in the C1 clusters. Subsequently, The DEGs across C1 and C2 subtypes were evaluated, yielding 4,266 upregulated and 573 downregulated genes. The results of GO, KEGG enrichment analysis showed that m7GRGs were mainly involved in the biological functions of myofibril, collagen-containing extracellular matrix, adhesive plaque, DNA replication, as well as the signaling pathways of adhesive plaque, cGMP PKG, cAMP, ECM receptor interaction, cell cycle in sarcomas. The findings of the GSEA enrichment study indicated that adhesion plaque, cell cycle checkpoint, DNA repair, TP53 transcriptional regulation, and EGFEGFR were among the probable biological processes and pathways implicated in sarcoma by m7GRGs. These biological functions and signaling pathways are intimately related to tumorigenesis and progression (Tang et al., 2017; Mai et al., 2021). Collagen subtype and modification may be used to predict the metastatic potential of these sarcomas (Eisinger-Mathason et al., 2013). Elevated cAMP signal transduction can be considered carcinogenic in osteosarcoma (Naviglio et al., 2006; Danieau et al., 2021). Further investigations are required to elucidate the signaling pathway of m7GRGs in sarcomas.

The correlation between the expression of C1 and C2 subtypes and various clinicopathological parameters was analyzed using TCGA database. There were significant differences between C1 group and C2 group in gender, race, and new tumor type. Prognostic m7GRGs (EIF4A1, EIF4G3, NCBP1, and WDR4) were identified based on the aforementioned expression level and prognosis analysis. Patients with sarcoma who had high levels of EIF4A1, EIF4G3, NCBP1, and WDR4 exhibited a lower OS rate. Eukaryotic initiation factor (eIF) 4F is a cytoplasmic complex consisting of three subunits: (i) eIF4E, a cap-binding protein that interacts with the m7G mRNA cap structure, (ii) eIF4G, as a protein scaffold, and (iii) eIF4A, an RNA helicase, utilized to remodel mRNA templates to facilitate ribosome recruitment (Pelletier and Sonenberg, 2019). EIF4A1 is the starting factor in the chromosome 17p13 gene map. It is also known as EIF4A (Pelletier and Sonenberg, 2019). EIF4A1 is associated with cancer cell malignancy, tumor-specific survival, and drug sensitivity (Xu et al., 2013; Liang et al., 2014; Modelska et al., 2015). circEIF4G3 is a novel form of circRNA that may be generated by reverse splicing of the EIF4G3 transcript (Zang et al., 2022). Circ-EIF4G3 is a closed loop structure, without 5‘end cap and 3′end of poly (A) tail, and is more stable (Wang et al., 2019b). Circ-EIF4G3 may contribute to the progression of gastric cancer, lung cancer, and other malignancies (Wang et al., 2019b; Huang et al., 2021). NCPB1 is a nuclear cap binding protein capable of heterodimerizing with NCPB2 and NCPB3 to produce a nuclear cap-binding complex (Gebhardt et al., 2015). Cap binding complex was discovered in HeLa cells for the first time. It may combine with the N7 methylguanine (m7G) “cap structure” of freshly transcribed mRNA and coordinate downstream RNA biogenesis, such as nuclear-cytoplasmic transport and recruitment of translation factors in the cytoplasm (Izaurralde et al., 1994; E Izaurralde et al., 1995; Calero et al., 2002). Huijun Zhang et al. found that NCBP1 may also accelerate lung cancer progression (Zhang et al., 2019a). WD repeat domain 4 (WDR4) is a member of the WD repeat protein family, which is associated with several aspects of cell development, such as cell cycle evolution, signal transduction, gene regulation, and apoptosis (Michaud et al., 2000; Rastegari et al., 2020; Ma et al., 2021). WDR4 performs a crucial part in a number of malignant cancers (Zeng et al., 2021).

The four distinctive genes were evaluated using univariate, multivariate, and LASSO Cox regression analysis (EIF4A1, EIF4G3, NCBP1, and WDR4). Then, the risk distribution analysis, ROC curve analysis and survival analysis were conducted. The results showed that high expression of EIF4A1, EIF4G3, NCBP1, and WDR4 elevated the risk score, and the high-risk group had a considerably shorter OS rate than the low-risk group. WDR4 and race were also identified as independent risk factors for sarcomas. An effective nomogram was constructed to predict the 1-year, 3-year, and 5-year survival rates of patients with sarcoma, indicating that WDR4 and race\ have a significant influence on the prevalence and prognosis of sarcomas.

As a vital part of the tumor microenvironment, immune cells played a crucial role in tumor progression (Thakkar et al., 2020). Previous studies found m7GRGs expression in ovarian cancer (OC) is significantly correlated with immune cells (involving CD4^+^ memory resting T cells, plasma cells, and Macrophages M1) (Zheng et al., 2022). In Hepatocellular carcinoma high m7G risk led to a decreased infiltration level of CD8^+^ T cells, whereas it increased the infiltration levels of Tregs and macrophages (Zhou et al., 2022). METTL1 expression was enhanced in HCC, accompanied by increased CD11b CD15 polymorphonuclear-myeloid-derived suppressor cells (PMN-MDSCs) and decreased CD8 T cells. Mechanistically, heat-mediated METTL1 upregulation enhanced TGF-β2 translation to form the immunosuppressive environment by induction of myeloid-derived suppressor cell (Zeng et al., 2022). In genetically engineered mouse models, the WDR4/Promyelocytic leukemia (PML) axis elevates intratumoral Tregs and M2-like macrophages and reduces CD8^+^ T cells to promote lung tumor growth. Our study identifies WDR4 as an oncoprotein that negatively regulates PML *via* ubiquitination to promote lung cancer progression by fostering an immunosuppressive and prometastatic tumor microenvironment, suggesting the potential of immune-modulatory approaches for treating lung cancer with aberrant PML degradation (Wang et al., 2017). The expression of eIF4E acts on mouse dendritic cells, resulting in increased activation of cytotoxic CD8 T cells *ex vitro* (Li et al., 2017c). In this study m7GRGs were associated with the degree of immune infiltration in sarcomas, which is another major conclusion of this study. EIF4G3 and NCBP1 were substantially negatively correlated with CD4^+^ T cells and dendritic cells. However, EIF4A1 and WDR4 were not significantly correlated with immune cell infiltration in sarcomas. A spearman analysis revealed that a high level of m7GRGs expression was negatively correlated with the majority of immune cells.

TMB and MSI were suggested as potential biomarkers for predicting the response of immunosuppressive agents at immune checkpoints (Dudley et al., 2016; Ritterhouse, 2019). In several tumor types, increased TMB was associated with the response to immunosuppressive agents (Ritterhouse, 2019). TMB and MSI scores of sarcomas were dramatically enhanced with the increase of EIF4G3, NCBP1, and WDR4 expression according to our findings. EIF4A1 improved the MSI score while having little impact on the TMB score. In addition, prognostic m7GRGs were positively or negatively correlated with a variety of chemotherapy drugs through the CDSC database. These findings may provide a novel potential therapeutic target for sarcomas.

Matrix components, such as CAFs and macrophages associated with tumors, plays a significant role in the onset and progression of cancer (Hanahan and Coussens, 2012). m7GRGs may upregulate prognostic CD4Tconv, Tprolif, CD8T, NK, DC, macrophages, and fibroblasts in sarcomas. In addition, prognostic m7GRGs are positively correlated with many markers of CAFs. The expression of prognostic m7GRGs is positively correlated with the infiltration of CAFs. Previous studies have shown that CAFs are highly invasive in recurrent osteosarcoma (Huang et al., 2022). Therefore, prognostic m7GRGs may influence the progression of sarcoma patients by altering the expression of CAFs, tumor-associated macrophages, and other immune cells in the TME.

Cuproptosis is a novel copper-dependent and regulated form of cell death that differs from the known way of cell death, in that copper may directly interact with the fatty acylation component of the TCA, resulting in protein toxicity stress and eventually cell death (Tsvetkov et al., 2022). Cuproptosis-related genes play an important role in the occurrence and development of various cancers, such as sarcoma and liver cancer (Zhang et al., 2022b; Han et al., 2022). This study revealed a tight association between m7GRGs and cuproptosis-associated genes in sarcomas. These two kinds of genes might jointly influence the occurrence and development of sarcomas, but further research is required to confirm this theory.

Four miRNA-mRNA regulatory axes and six lncRNA-miRNA-mRNA regulatory axes were also constructed, including has-miR-28-5p/EIF4A1, has-miR-145-5p/EIF4G3, has-miR-489-3p/NCBP1, and has-miR-28-5p/WDR4, CSA 9-has-miR-195-5p-WDR4, LINC00511-has-miR-195-5p-WDR4, LINC00922-has-miR-195-5p-WDR4, MEG3-has-miR-195-5p-WDR4, MEG8-has-miR-195-5p-WDR4, and SNHG16-has-miR-195-5p-WDR4. The miR-185-3p/E2F1 axis was regulated by LINC00511 to promote the occurrence and progression of osteosarcoma (Xu et al., 2020). The prognosis of many cancers was predicted by SNHG16 (Zhang et al., 2019b). MiR-128-5p participated in the progression of colorectal cancer (Si et al., 2021). Our study revealed that these miRNAs and lncRNAs were related to the prognosis of sarcoma patients. All these pieces of evidence suggested that these regulatory axes might play an important role in the progression of sarcomas. Our study also has certain limitations. First, the sample size of the control group was small. In addition, further research should be conducted to corroborate this conclusion.

## 5 Conclusion

In conclusion, dataset analysis revealed that the expression of m7GRGs (EIF4A1, EIF4G4, NCBP1, and WDR4) was strongly associated with clinicopathological characteristics of SARC. In addition, the relationships between the m7GRGs and tumor immune microenvironment, immunotherapy and chemotherapy response were preliminarily ascertained. Four miRNA-mRNA regulatory axes and six lncRNA-miRNA-mRNA regulatory axes were also identified, which may play an important role in the progress of sarcomas and may serve as potential diagnostic biomarkers and therapeutic targets for sarcomas. It is plausible to hypothesize that our study may provide valuable insights into clinical decision-making and individualized therapy regimens as a foundation for future research.

## Data Availability

The datasets are available in TCGA database (https://portal.gdc.cancer.gov/), GDSC database (https://www.cancerrxgene.org/), GeneMANIA (http://www.genemania.org), GSEA (http://software.broadinstitute.org/gsea/index.jsp), Human Protein Atlas database (https://www.proteinatlas.org), cBioPortal (http://www.cbioportal.org/), GDSC database (https://www.cancerrxgene.org/), TISIDB (http://cis.hku.hk/TISIDB), TISCH database (http://tisch.comp-genomics.org/), ENCORI database (http://starbase.sysu.edu.cn/), miRNet database (http://www.mirnet.ca/), RNA22 database (https://cm.jefferson.edu/rna22/interactive) as well as TIMER database (https://cistrome.shinyapps.io/timer/).
