# Supplementary material for: Identification and verification of m7G-Related genes as biomarkers for prognosis of sarcoma
Source: Front Genet. 2023 Feb 3;14:1101683. doi: 10.3389/fgene.2023.1101683 (PMC9935680; doi:10.3389/fgene.2023.1101683)
Supplement: Supplementary file 1 [file DataSheet1.PDF]

SUPPLEMENTAL DATA

Table S1. Presentation of part of the GO and KEGG pathway enrichment analysis for the DEGs associated with cancer genesis and progress

Ontology	ID	Description	Gene Ratio	Bg Ratio	p value	p. adjust	q value
BP	GO:0030239	myofibril assembly	18/989	73/18670	3.20e-08	1.67e-05	1.49e-05
BP	GO:0034329	cell junction assembly	33/989	241/18670	5.39e-07	2.16e-04	1.93e-04
BP	GO:0006260	DNA replication	34/989	274/18670	3.53e-06	0.001	0.001
BP	GO:1901987	regulation of cell cycle phase transition	50/989	480/18670	3.94e-06	0.001	0.001
BP	GO:0140014	mitotic nuclear division	32/989	264/18670	1.10e-05	0.003	0.002
BP	GO:0000075	cell cycle checkpoint	28/989	216/18670	1.11e-05	0.003	0.002
BP	GO:0031589	cell-substrate adhesion	39/989	354/18670	1.26e-05	0.003	0.002
BP	GO:0030198	extracellular matrix organization	40/989	368/18670	1.34e-05	0.003	0.002

Ontology	ID	Description	Gene Ratio	Bg Ratio	p value	p. adjust	q value
CC	GO:0043292	contractile fiber	51/1023	234/19717	1.17e-18	3.61e-16	2.86e-16
CC	GO:0030016	myofibril	48/1023	224/19717	2.54e-17	5.24e-15	4.16e-15
CC	GO:0030017	sarcomere	44/1023	204/19717	4.24e-16	6.57e-14	5.21e-14
CC	GO:0062023	collagen-containing extracellular matrix	57/1023	406/19717	6.99e-12	6.19e-10	4.91e-10
CC	GO:0030055	cell-substrate junction	54/1023	412/19717	3.44e-10	2.67e-08	2.12e-08
CC	GO:0005925	focal adhesion	52/1023	405/19717	1.53e-09	1.05e-07	8.34e-08
CC	GO:0005924	cell-substrate adherens junction	52/1023	408/19717	1.98e-09	1.23e-07	9.74e-08
CC	GO:0000775	chromosome, centromeric region	31/1023	193/19717	1.95e-08	1.01e-06	7.99e-07
MF	GO:0003779	actin binding	65/979	431/17697	1.31e-13	1.18e-10	1.09e-10
MF	GO:0005201	extracellular matrix structural constituent	26/979	163/17697	1.02e-06	4.60e-04	4.24e-04

MF	GO:0008179	adenylate cyclase binding	6/979	13/17697	3.46e-05	0.010	0.010
MF	GO:0015631	tubulin binding	37/979	336/17697	5.23e-05	0.012	0.011
MF	GO:0042393	histone binding	24/979	197/17697	2.35e-04	0.030	0.028
MF	GO:0005518	collagen binding	12/979	67/17697	2.74e-04	0.031	0.029
MF	GO:0004842	ubiquitin-protein transferase activity	38/979	382/17697	3.44e-04	0.034	0.032
MF	GO:0003887	DNA-directed DNA polymerase activity	7/979	27/17697	5.19e-04	0.047	0.043
KEGG	hsa04270	Vascular smooth muscle contraction	22/428	135/8076	2.05e-06	3.23e-04	2.68e-04
KEGG	hsa04510	Focal adhesion	28/428	201/8076	2.22e-06	3.23e-04	2.68e-04
KEGG	hsa04022	cGMP-PKG signaling pathway	24/428	167/8076	6.92e-06	6.71e-04	5.56e-04
KEGG	hsa04024	cAMP signaling pathway	26/428	216/8076	6.96e-05	0.005	0.004
KEGG	hsa05205	Proteoglycans in cancer	25/428	205/8076	7.74e-05	0.005	0.004

KEGG	hsa04512	ECM-receptor interaction	14/428	88/8076	1.95e-04	0.009	0.007
KEGG	hsa04810	Regulation of actin cytoskeleton	25/428	218/8076	2.08e-04	0.009	0.007
KEGG	hsa04110	Cell cycle	17/428	124/8076	2.71e-04	0.010	0.008

Table S2. Gene sets enriched in phenotype high.

Gene set name	NES	p. adjust	FDR
REACTOME_CELL_CYCLE_CHECKPOINTS	1.615	0.004	0.003
REACTOME_DNA_REPAIR	1.574	0.004	0.003
WP_FOCAL_ADHESION	1.331	0.026	0.019
WP_EGFEGFR_SIGNALING_PATHWAY	1.459	0.014	0.01
REACTOME_TRANSCRIPTIONAL_REGULATION_BY_TP53	1.447	0.004	0.003

NES: normalized enrichment score; FDR: false discovery rate. Gene sets with p-value <0.05 and FDR q-value <0.25 are considered as significant.

Table S3. The clinical characters of sarcoma patients in TCGA cohort.

Clinical characters	Number
Alive	161

Dead	99
Mean (SD)	60.6 (14.7)
FEMALE	141
MALE	119
ASIAN	6
BLACK	18
WHITE	227
Metastasis	68
Primary	8
Recurrence	49
Non-radiation	140
Radiation	74
Neoadjuvant	1
No neoadjuvant	259
Ancillary: Chemotherapy	1
Ancillary: Chemotherapy: Hormone Therapy	1
Chemotherapy	59
Chemotherapy:	1
Chemotherapy: Hormone Therapy	2
Chemotherapy: Immunotherapy	3
Chemotherapy: Immunotherapy: Targeted Molecular therapy	1

Chemotherapy: Targeted Molecular therapy	3
Hormone Therapy	1

Table S4. Correlation analysis between lncRNA and hsa-miR-195-5p or lncRNA and WDR4 in GC determined by starBase and TCGA databases.

LncRNA	miRNA	R value	p value
LINC02434	hsa-miR-195-5p	-0.184	2.85E-03
CASC9	hsa-miR-195-5p	-0.125	4.34E-02
LINC00943	hsa-miR-195-5p	-0.133	3.22E-02
MEG3	hsa-miR-195-5p	-0.217	4.24E-04
MEG8	hsa-miR-195-5p	-0.290	1.95E-06
LINC00922	hsa-miR-195-5p	-0.171	5.75E-03
LINC00511	hsa-miR-195-5p	-0.122	4.89E-02
SNHG16	hsa-miR-195-5p	-0.313	2.43E-07
SMIM25	hsa-miR-195-5p	-0.169	6.18E-03
MIR503HG	hsa-miR-195-5p	-0.162	8.75E-03
**LncRNA**	**mRNA**	**R value**	**p value**
LINC02434	WDR4	0.146	1.76E-02
CASC9	WDR4	0.175	4.40E-03
LINC00943	WDR4	0.269	9.80E-06
MEG3	WDR4	0.163	8.15E-03

MEG8	WDR4	0.159	9.70E-03
LINC00922	WDR4	0.249	4.41E-05
LINC00511	WDR4	0.240	8.25E-05
SNHG16	WDR4	0.344	1.02E-08
SMIM25	WDR4	0.253	3.22E-05
MIR503HG	WDR4	0.214	4.84E-04

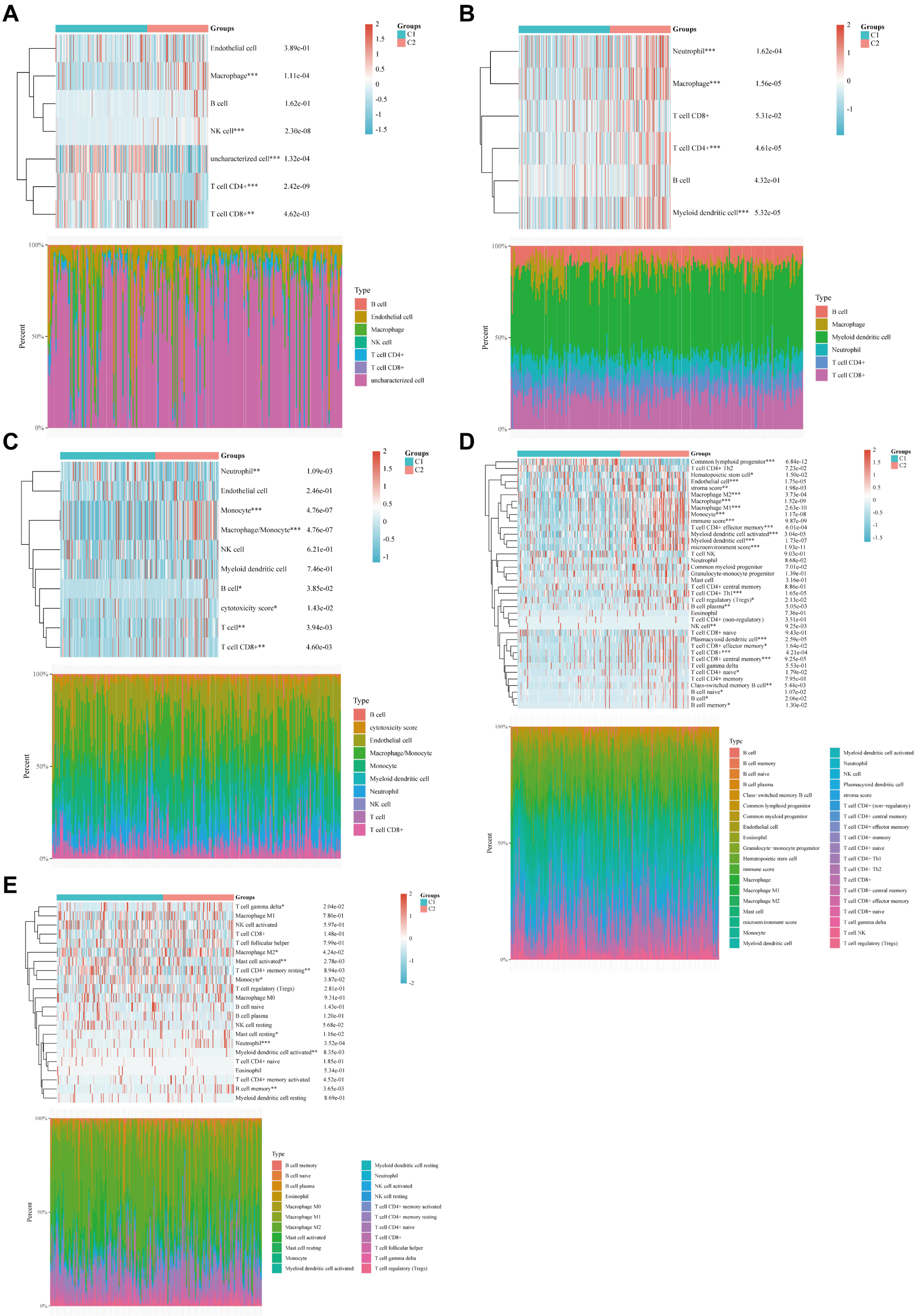

Figure S1. Relationship between m7GRG expression and immune infiltration in the

TME. (A-E) Comparison of immune scores between C1 and C2 subtypes in TCGA (CIBERSORT, TIMER, EPIC, MCP-counter, and xCell).

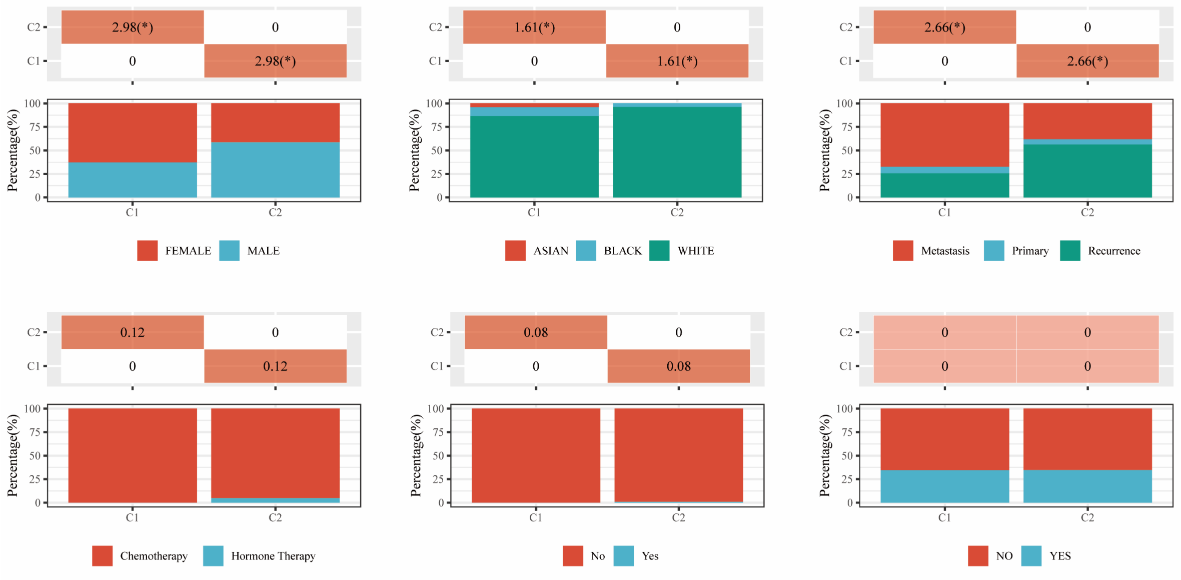

Figure S2. Clinicopathological characteristics of C1and C2 in sarcomas.

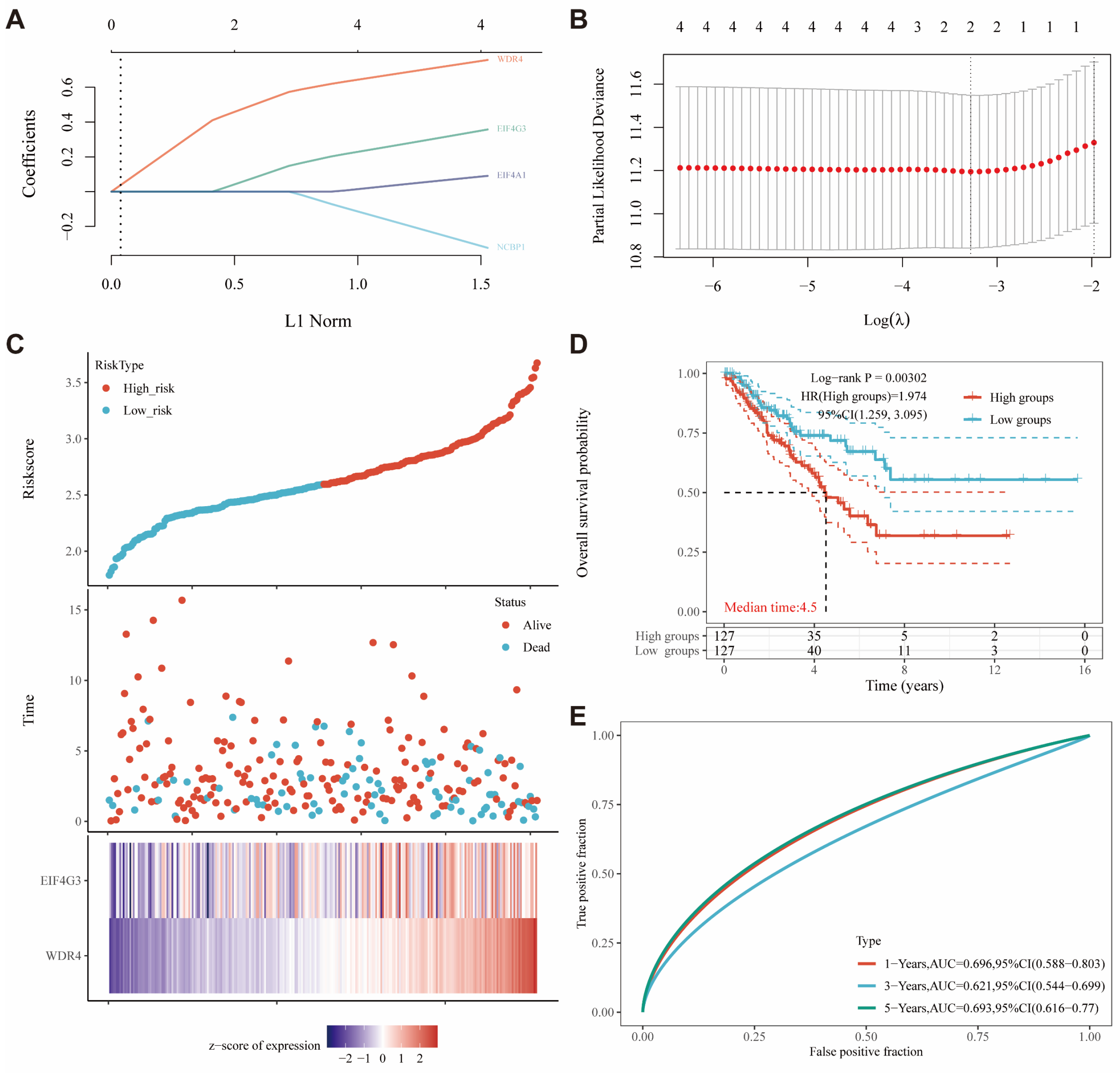

Figure S3. Construction of a prognostic CRG model (DSS). (A) LASSO coefficient profiles of prognostic m7GRGs. (B) Plots of the ten-fold cross-validation error rates. (C) Distribution of the risk score, survival status, and the expression of prognostic m7GRGs in sarcomas. (D-E) DSS curves of sarcoma patients in the high-/low-risk group and the ROC curve for measuring the predictive value.

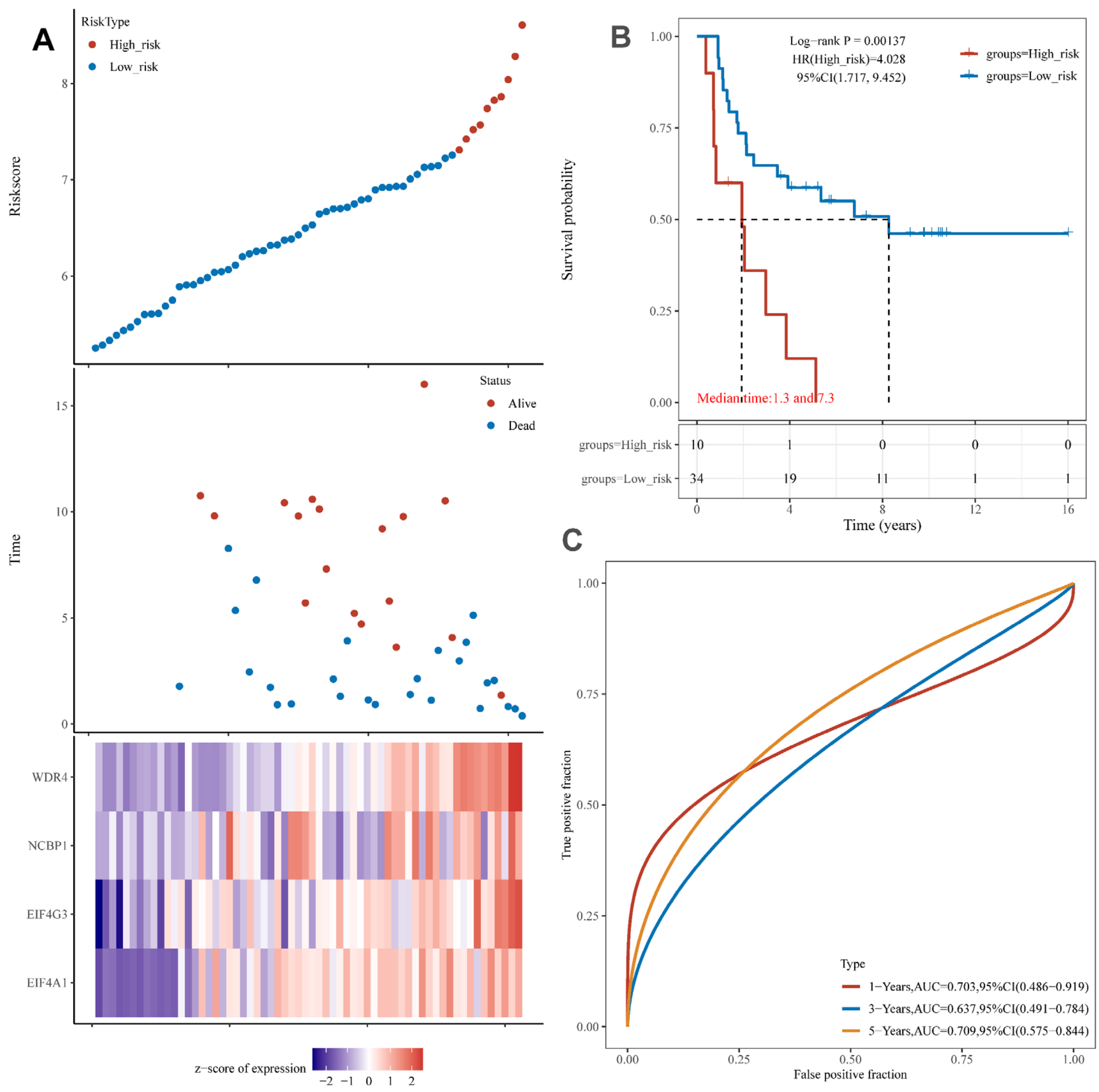

Figure S4. The validation of risk scoring model in GEO data set (GSE17674). (A) Risk score map of each SARC patient and distribution map of survival time and m7GRGs expression of each SARC patient; (B) The total survival curve of SARC patients in high/low risk group among SARC patients; (C) Time-dependent ROC curve of OS in the period of 1, 3 and 5 years for m7GRGs.

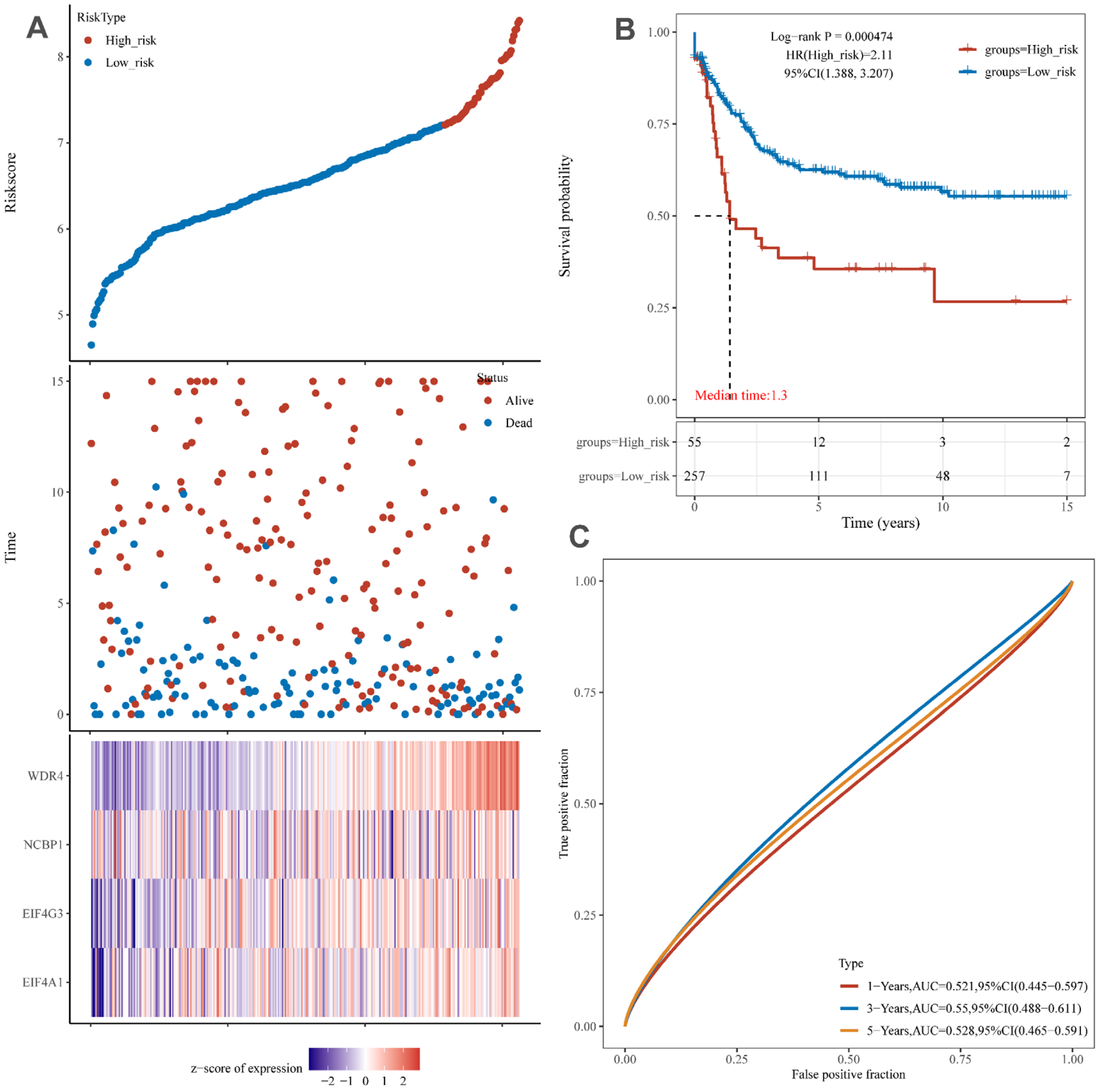

Figure S5. The validation of risk scoring model in GEO data set (GSE71118). (A) Risk score map of each SARC patient and distribution map of survival time and m7GRGs expression of each SARC patient; (B) The total survival curve of SARC patients in high/low risk group among SARC patients; (C) Time-dependent ROC curve of OS in the period of 1, 3 and 5 years for m7GRGs.

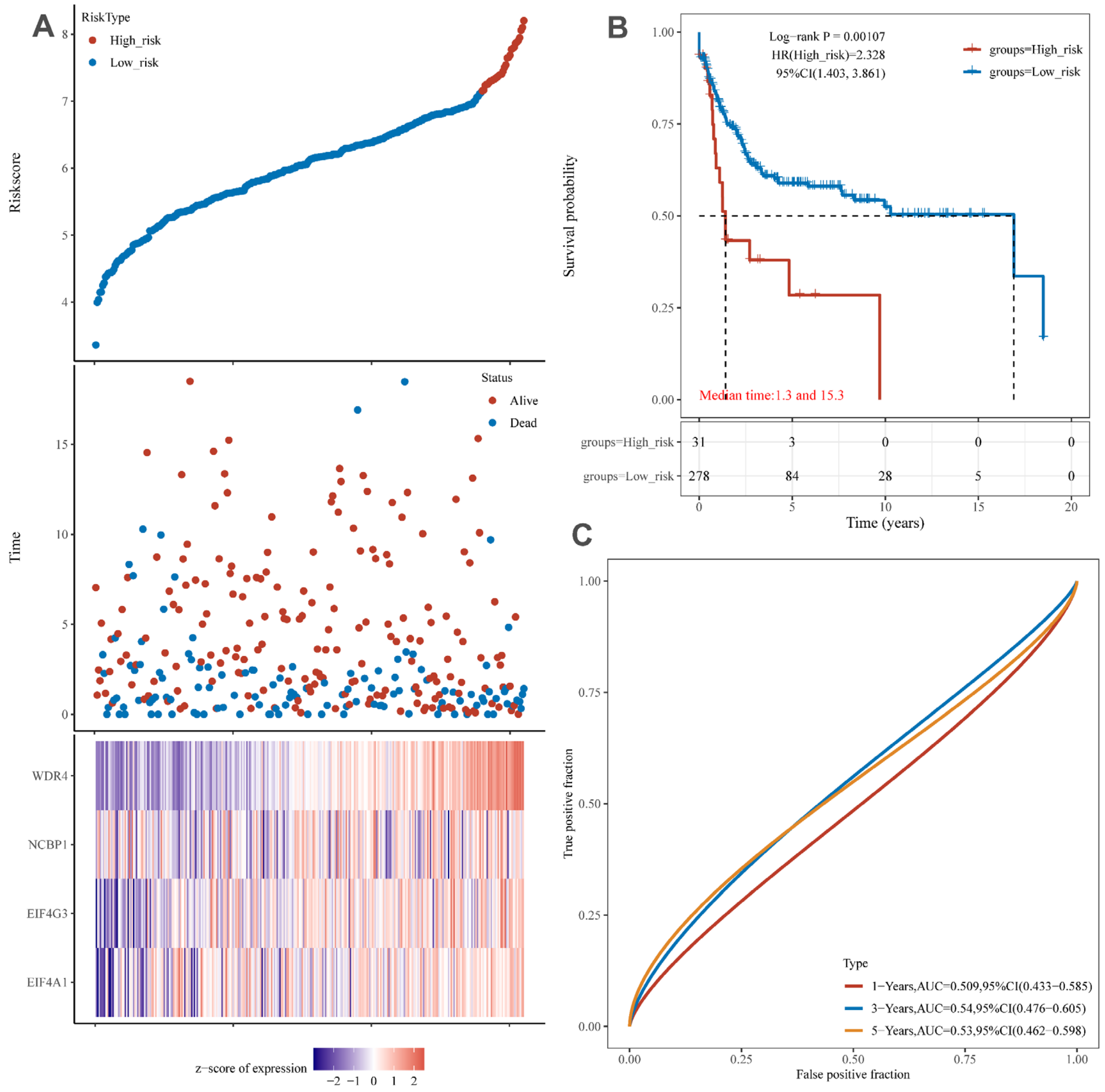

Figure S6. The validation of risk scoring model in GEO data set (GSE21050). (A) Risk score map of each SARC patient and distribution map of survival time and m7GRGs expression of each SARC patient; (B) The total survival curve of SARC patients in high/low risk group among SARC patients; (C) Time-dependent ROC curve of OS in the period of 1, 3 and 5 years for m7GRGs.

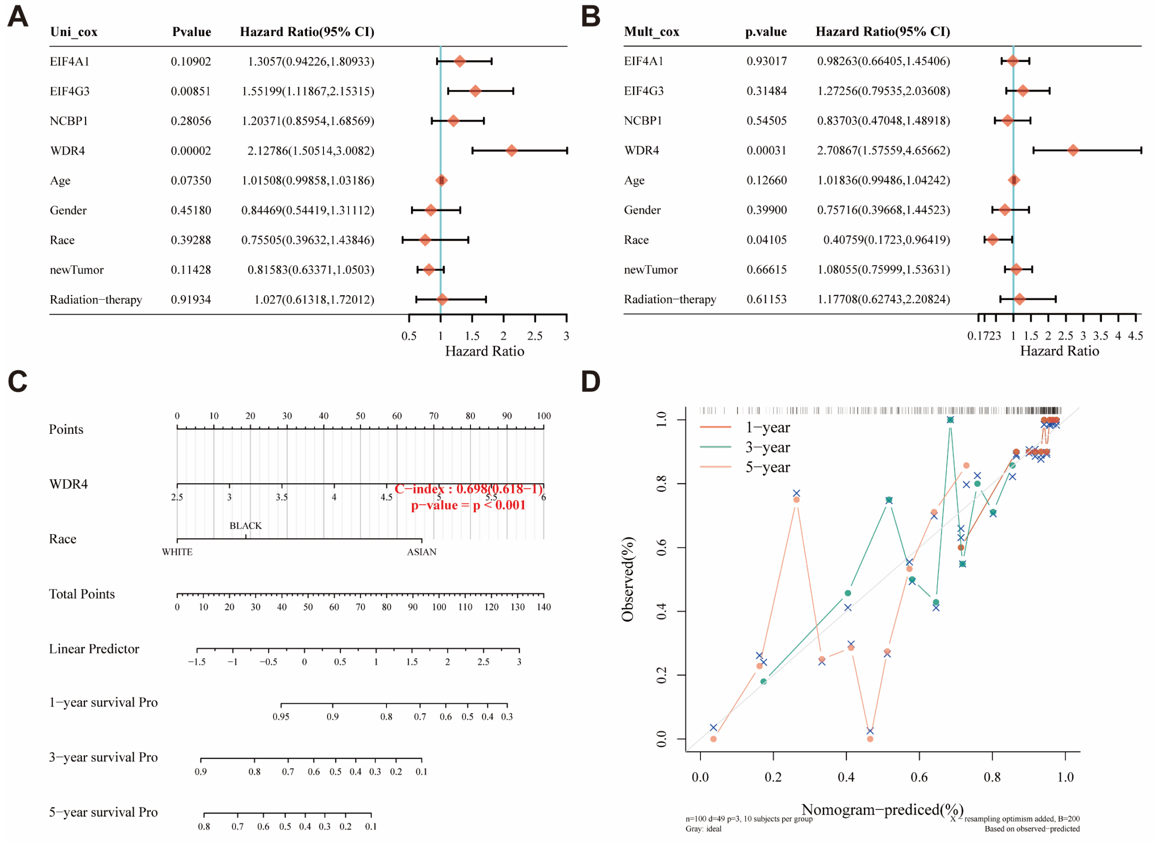

Figure S7. Construction of a predictive nomogram (DSS). (A, B) Hazard ratios and P value of the constituents involved in univariate and multivariate Cox regression analysis considering the clinical information and prognostic m7GRGs in sarcomas. (C) Nomogram to predict the 1-year, 3-year and 5-year DSS rate of sarcoma patients. (D) Calibration curve for the DSS nomogram model in the discovery group. The dashed diagonal line represents the ideal nomogram.

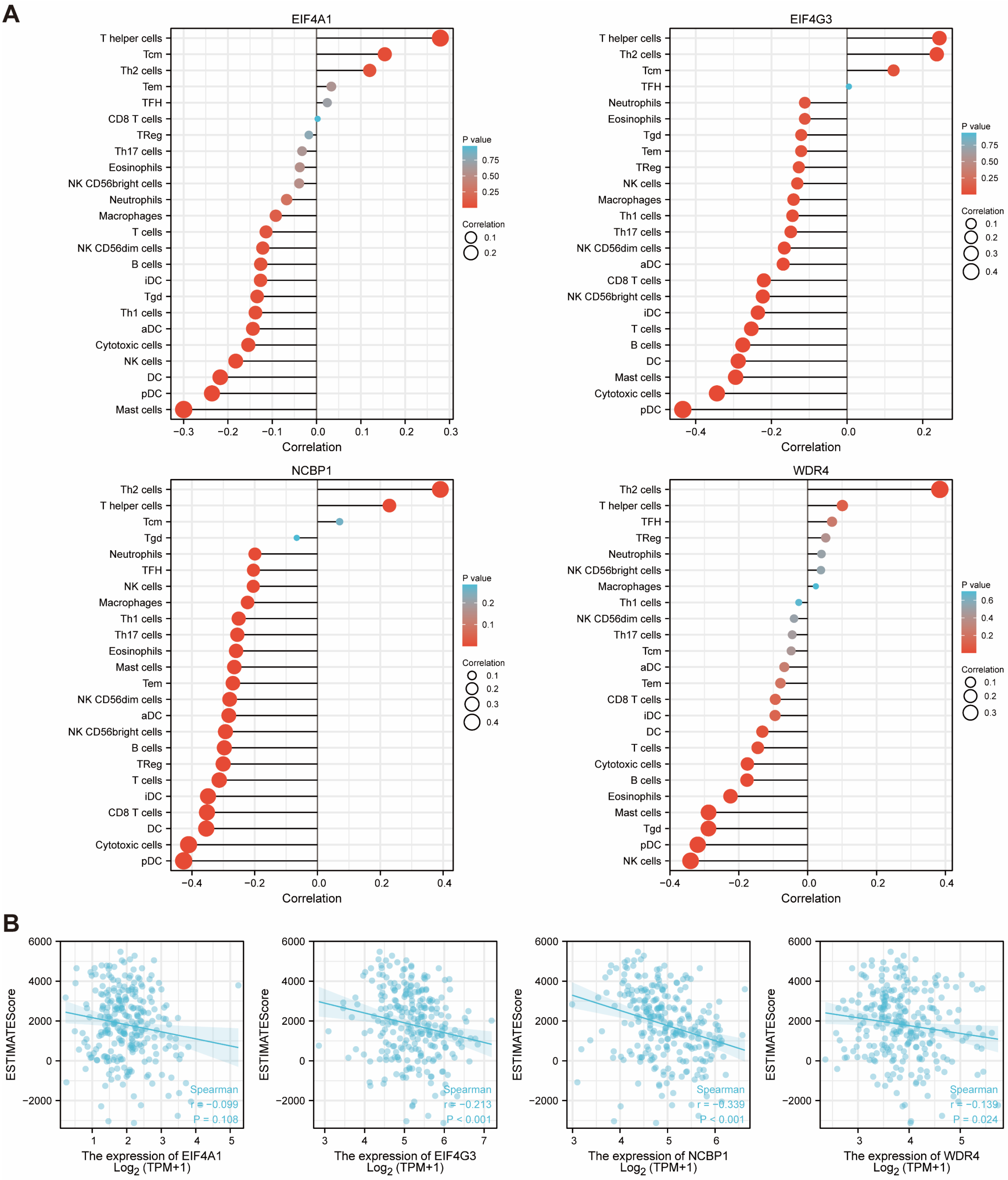

Figure S8. Figure (A) Correlation between prognostic m7GRGs and immune cell infiltration; Figure (B) Correlation between prognostic m7GRGs and ESTIMATEscore.

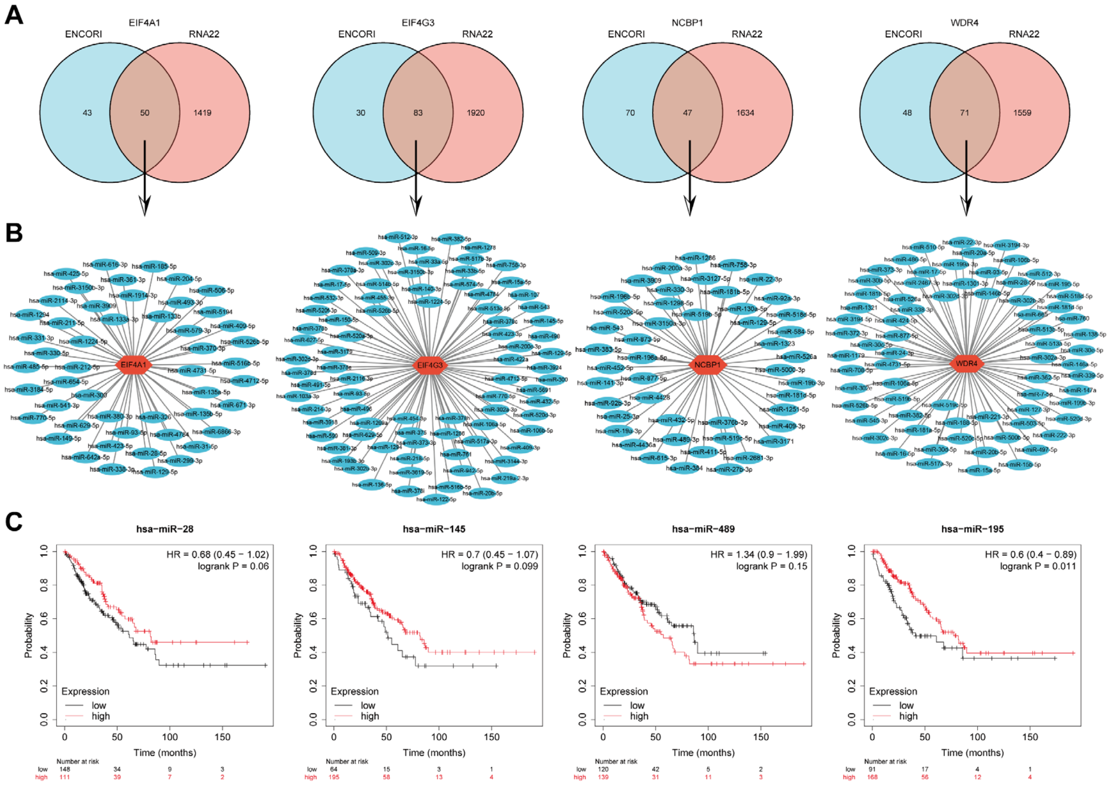

Figure S9. Identification of the potential miRNAs related to the prognosis of sarcomas. (A) Potential predicted miRNAs associated with m7GRGs by ENCORI and RNA22 databases. (B) Potential mRNA-miRNA gene networks constructed using Cytoscape software. (C) Expression and prognostic value of miRNAs (hsa-miR-195-5p).

The abbreviations of m7GRGs, CAF, CRGs and immune-related checkpoints in this study.

m7GRGs Abbreviation	Full name
AGO2	Protein argonaute-2
CYFIP1	Cytoplasmic FMR1-interacting protein 1
DCP2	m7GpppN-mRNA hydrolase
DCPS	m7GpppX diphosphatase
EIF3D	Eukaryotic translation initiation factor 3 subunit D
EIF4A1	Eukaryotic initiation factor 4A-I
EIF4E	Eukaryotic translation initiation factor 4E
EIF4E2	Eukaryotic translation initiation factor 4E type 2
EIF4E3	Eukaryotic translation initiation factor 4E type 3
EIF4G3	Eukaryotic translation initiation factor 4E type 3
GEMIN5	Gem-associated protein 5
IFIT5	Interferon-induced protein with tetratricopeptide repeats 5
LARP1	La-related protein 1
LSM1	U6 snRNA-associated Sm-like protein LSm1
METTL1	tRNA (guanine-N(7)-)-methyltransferase
NCBP1	Nuclear cap-binding protein subunit 1
NCBP2	Nuclear cap-binding protein subunit 2
NCBP2L	Nuclear cap-binding protein subunit 2-like

NCBP3	Nuclear cap-binding protein subunit 3
NSUN2	RNA cytosine C(5)-methyltransferase NSUN2
NUDT10	Diphosphoinositol polyphosphate phosphohydrolase 3-alpha
NUDT11	Diphosphoinositol polyphosphate phosphohydrolase 3-beta
NUDT16	U8 snoRNA-decapping enzyme
NUDT3	Diphosphoinositol polyphosphate phosphohydrolase 1
NUDT4	Diphosphoinositol polyphosphate phosphohydrolase 2
SNUPN	Snurportin-1
WDR4	tRNA (guanine-N(7)-)-methyltransferase non-catalytic subunit WDR4

**CAF Abbreviation**	**Full name**
PDGFRA	Platelet-derived growth factor receptor alpha
PDGFRB	Platelet-derived growth factor receptor beta
S100A4	Protein S100-A4
FAP	Prolyl endopeptidase FAP
VIM	Vimentin
COL11A1	Collagen alpha-1(XI) chain
PDPN	Podoplanin
ITGA11	Integrin alpha-11

POSTN	Periostin
PDGFB	Platelet-derived growth factor subunit B
WNT5A	Protein Wnt-5a
PDGFD	Platelet-derived growth factor D
COL3A1	Collagen alpha-1(III) chain
DACH1	Dachshund homolog 1

**CRGs Abbreviation**	**Full name**
FDX1	Adrenodoxin, mitochondrial
LIAS	Lipoyl synthase, mitochondrial
LIPT1	Lipoyltransferase 1, mitochondrial
DLD	Dihydrolipoyl dehydrogenase, mitochondrial
DLAT	Dihydrolipoyllysine-residue acetyltransferase component of pyruvate dehydrogenase complex, mitochondrial
PDHA1	Pyruvate dehydrogenase E1 component subunit alpha, somatic form, mitochondrial
PDHB	Pyruvate dehydrogenase E1 component subunit beta, mitochondrial
MTF1	Metal regulatory transcription factor 1
GLS	Glutaminase kidney isoform, mitochondrial
CDKN2A	Tumor suppressor ARF
SLC31A1	High affinity copper uptake protein 1

ATP7B	Copper-transporting ATPase 2

**immune-related checkpoints Abbreviation**	**Full name**
SIGLEC15	Sialic acid-binding Ig-like lectin 15
TIGIT	T-cell immunoreceptor with Ig and ITIM domains
CD274	Programmed cell death 1 ligand 1
HAVCR2	Hepatitis A virus cellular receptor 2
PDCD1	Programmed cell death protein 1
CTLA4	Cytotoxic T-lymphocyte protein 4
LAG3	Lymphocyte activation gene 3 protein
PDCD1LG2	Programmed cell death 1 ligand 2
